# Data-driven simulation and characterisation of gold nanoparticle melting

**DOI:** 10.1038/s41467-021-26199-7

**Published:** 2021-10-18

**Authors:** Claudio Zeni, Kevin Rossi, Theodore Pavloudis, Joseph Kioseoglou, Stefano de Gironcoli, Richard E. Palmer, Francesca Baletto

**Affiliations:** 1 Department of Physics, King’s College London, London, WC2R 2LS UK; 2 College of Engineering, Swansea University, Bay Campus, Fabian Way, Swansea, SA1 8EB UK; 3 Department of Physics, Aristotle University of Thessaloniki, Thessaloniki, GR-54124 Greece; 4 Present Address: International School for Advanced Studies, Via Bonomea, 265, 34136 Trieste, Italy; 5 Present Address: International School for Advanced Studies, Via Bonomea, 265, 34136 Trieste, Italy; 6 Present Address: Laboratory of Nanochemistry, Institute of Chemistry and Chemical Engineering, Ecole Polytechnique Fédérale de Lausanne, Lausanne, Switzerland; 7 Present Address: Department of Physics, Aristotle University of Thessaloniki, Thessaloniki, GR-54124 Greece; 8 Present Address: DIPC, Paseo Manuel de Lardizabal, 20018 San Sebastian, Spain

**Keywords:** Nanoparticles, Atomistic models, Computational methods

## Abstract

The simulation and analysis of the thermal stability of nanoparticles, a stepping stone towards their application in technological devices, require fast and accurate force fields, in conjunction with effective characterisation methods. In this work, we develop efficient, transferable, and interpretable machine learning force fields for gold nanoparticles based on data gathered from Density Functional Theory calculations. We use them to investigate the thermodynamic stability of gold nanoparticles of different sizes (1 to 6 nm), containing up to 6266 atoms, concerning a solid-liquid phase change through molecular dynamics simulations. We predict nanoparticle melting temperatures in good agreement with available experimental data. Furthermore, we characterize the solid-liquid phase change mechanism employing an unsupervised learning scheme to categorize local atomic environments. We thus provide a data-driven definition of liquid atomic arrangements in the inner and surface regions of a nanoparticle and employ it to show that melting initiates at the outer layers.

## Abstract (web summary)

Efficient theoretical methods for the structural analysis of nanoparticles are very much needed. Here the authors demonstrate the use of machine-learning force fields and of a data-driven approach to study the thermodynamical stability and elucidate the melting process of gold nanoparticles.

## Introduction

Gold (Au) nanoparticles (NPs) find widespread application in many technological areas, such as in optics^1,2^, nanomedicine^3,4^ and catalysis^5–9^. As all chemo-physical properties of Au NPs depend on their shape, the analysis of their structural stability has attracted a lot of attention in the past years. A deep understanding of the liquid–solid phase change mechanisms of Au NP, also accounting for surface rearrangements, may, in particular, be crucial for catalytic applications, where the reaction conditions often demand the nanocatalysts to work at high temperatures while preserving their size and shape.

Numerical simulations can, in principle, offer a platform to investigate and characterize phase change mechanisms of NPs at an atomistic level. However, two long-standing challenges must be overcome to improve the numerical predictions of NPs’ thermal stability. The first concerns the difficulty of defining an unbiased characterization of the phase change mechanism. Indeed, the identification of order parameters to characterize solid–liquid phase changes at the nanoscale is an active topic of debate with a long tradition^10–13^. Widely used methods often rely on chemical-intuition and heuristic approaches, and can therefore lead to descriptive order parameters, which are neither fully general nor robust to parameter tuning. For example, changes in the first neighbour distance distribution affect the definition of coordination number too drastically^14,15^, and little research has been carried out on the characterization of local atomic environments peculiar of NP’s surface atoms.

The second challenge is related to the development of accurate and fast interparticle potentials, which reproduce the complexity of the NPs’ energy landscape. In so far, atomistic modelling methods have offered a strict trade-off between computational speed and accuracy. While simulations based on electronic structure methods, such as density functional theory (DFT), provide quantitative accuracy, their computational cost severely limits the capabilities to generate dynamical trajectories of large systems and for long times. On the contrary, large systems and long simulation timescales are easily accessible when employing semi-empirical potentials. Nevertheless, such methods do not necessarily provide a quantitative insight on the chemistry of NPs’ phase changes^16^ because their analytical functional form limits their predictive power and flexibility. Furthermore, these potentials are often fitted to bulk properties, which poses an additional limit to their accuracy when simulating nanoscale systems^17^.

In this work, we tackle these two challenges by adopting data-driven methods to generate an accurate and efficient description of interatomic potentials, and by developing an automated routine that classifies the atomic environments observed during Au NPs’ phase change. To obtain long, i.e., hundreds of nanoseconds, and accurate trajectories during melting of Au NPs of variable sizes, we develop a set of machine-learning force fields (ML-FFs)^18–25^ using the innovative framework of mapped Gaussian processes^26–28^. ML-FFs can approximate the force-energy predictions yielded by the reference DFT method they are trained upon while being many orders of magnitude faster to compute. Here, we train ML-FFs on local-density approximation (LDA)-DFT data and revised Perdew-Burke-Ernzerhof (rPBE)-DFT data, and contrast our results with experimental results and with predictions found using a semi-empirical interatomic potential. To characterize the melting kinetics, we adopt an unsupervised ML clustering scheme that discriminates in an automatic fashion locally liquid from locally solid environments, surface from inner environments and high-coordination from low-coordination surface environments. We then obtain a route to estimate the NPs melting temperature by monitoring the relative population of liquid atoms in the NP and the melting mechanism by recording the spatial distribution of locally liquid environments as a function of temperature. We employ these data-driven tools to study the melting of Au NPs with diameters between 1 and 6 nm, and various initial geometrical shapes. We univocally show that melting initiates in the outermost layer of Au NPs first, and occurs in the NPs’ core second.

## Results

### Machine-learning force fields

To construct a training database, we extract seven random de-correlated frames from ab initio molecular dynamics trajectories where an Au NP containing 309 atoms (~2 nm of diameter) with an initial face-centred cubic (FCC) morphology undergoes melting. We calculate, for each frame, forces and energies at DFT LDA and DFT generalized gradient approximation (GGA)-rPBE levels, and utilize a 2 + 3-body mappable Gaussian process regression (GPR) framework^26,27^ to fit two ML-FFs, one for each DFT method; further detail is provided in the ‘Methods’ and in the Supplementary Methods. When training on 2100 local atomic environments, our ML-FFs incur in a mean absolute error (MAE) on the force components of 0.09 ± 0.04 eV/Å (LDA ML-FF) and 0.07 ± 0.03 eV/Å (rPBE ML-FF), and in an MAE on the atomic energy differences of 2.65 ± 2.02 meV/atom (LDA ML-FF) and 1.98 ± 1.76 meV/atom (rPBE ML-FF), on validation sets disjointed from the training sets. The reported accuracy is comparable to the ones quoted in previous studies^29–35^ and is deemed satisfactory. This training dataset, albeit small, contains a heterogeneous set of local atomic environments, as shown in Supplementary Fig. 1, and we, therefore, consider it to be representative for Au NPs in the size range of interest.

We test the accuracy of the two ML-FFs on a more complex dataset, which encompasses NPs’ architectures of different sizes and shapes (see Supplementary Methods). Supplementary Table 1 reports the MAEs on force components and atomic energies incurred by each of the ML-FFs developed on these validation datasets. The MAEs on force components are again consistently around 0.1 eV/Å, and the MAEs on atomic energy differences are consistently lower than 10 meV/atom. The ML-FFs are, therefore, considered accurate enough and, more importantly, transferable across different NPs’ sizes and shapes. This holds regardless of the DFT level of theory used to train the ML-FF (GGA PBE and LDA) and its implementation (Vienna Ab initio Simulation Package (VASP) projector-augmented wave and CP2K Gaussian plane wave).

When validating the ML-FFs against the experimental bulk cohesive energy (Supplementary Fig. 4), we observe that LDA (rPBE)-based ML-FF overestimates (underestimates) this quantity. We then adopt a parametric mixing of the two ML-FFs (see also the ‘Methods’ section) and generate a third ML-FF, labelled hybrid, which, by construction, has cohesive energy in the bulk phase that matches the experimental one. The 2- and 3-body FFs forming the three ML-FFs present some noticeable differences; in Supplementary Figure 5, we show how the LDA ML-FF is more bound and stiffer than the rPBE ML-FF, and how the hybrid ML-FF has, as expected, a shape that is in-between one of the other two ML-FFs.

### Phase change characterization

Following the successful training and validation of our ML-FFs, we employ them to study the size-dependent melting temperature of Au NPs. We consider NPs whose diameter ranges from 1 to 6 nm, corresponding to NPs containing 147, 309, 561, 923, 2869, and 6266 atoms. We sample the NPs’ evolution in a temperature range between 400 and 1600 K when subject to a heating rate of 20 K/ns. We also test 5 and 10 K/ns heating rates and do not observe significant changes in the melting temperature estimate (see Supplementary Fig. 6). For each NP size and ML-FF, we simulate Au NPs for a total of 2.4 ms, a time length not accessible to electronic structure calculations, even for the largest state-of-the-art computational facilities. We refer the interested reader to the ‘Methods’ section for further details on the numerical set-up used to perform the simulations.

**Fig. 1:**
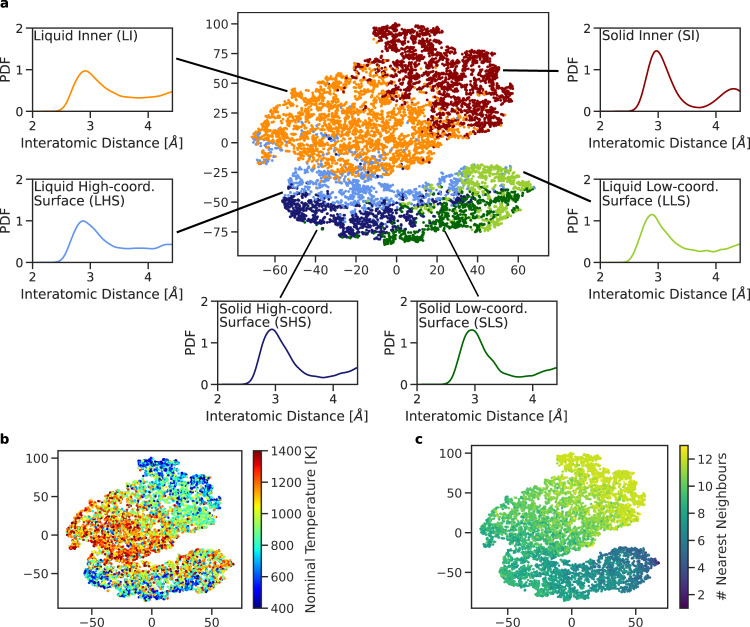
Features of the six classes of local atoms environments identified through clustering. Visualization of the hierarchical k-means clustering results for MD simulations of Au nanoparticles with 147, 309, 561, 923, 2869, and 6266 atoms, carried out using the ML-FF trained on rPBE-DFT data. **a** First and second component (*x*- and *y*-axis) of the t-sne projection of the atomic expansion coefficients of 10^4^ local atomic environments randomly sampled from melting MD simulations. The colours label the six classes assigned by the hierarchical *k*-means clustering algorithm, as defined in the main text. The normalized average pair-distance distribution function (PDF) belonging to each class is shown and coloured accordingly. **b**, **c** Same t-sne projection as in (**a**). In (**b**), the colours indicate the nominal simulation temperature at which the local atomic environment was taken from; in (**c**), the number of nearest neighbours (#NNs) was computed using a cut-off of 3.6 Å.

To characterize the solid–liquid phase transition, while distinguishing between surface and inner melting, we adopt an unsupervised ML approach that hinges on a small database of configurations randomly extracted from the phase change trajectories we simulated and a local atomic density representation. In particular, we employ a modified version^36^ of the 3-body local atomic cluster expansion descriptor^37^ to associate a 40-dimensional set of features to each atom. We then exploit a hierarchical *k*-means clustering scheme to isolate six classes of local atomic environments (see also the ‘Methods’ and Supplementary Methods). This clustering scheme labels the local atomic environments as being in the solid or liquid phase, and as belonging to the inner, high-coordination surface and low-coordination surface motifs. As illustrated in panels b and c of Fig. 1 and Supplementary Figs. 6 and 13, both the number of nearest neighbours (#NNs) within a predefined cut-off and the nominal MD simulation temperature at which these are sampled correlate with the labels assigned by the clustering algorithm.

The six local atomic environment classes are showcased in Fig. 1 and Supplementary Figs. 8 and 9 can be characterized from the number of neighbours within a given cut-off (here taken as 3.50 Å for the LDA ML-FF, 3.75 Å for the rPBE ML-FF and 3.60 Å for the hybrid ML-FF) they display, and from features in their pair-distance distribution function (PDF). In detail:

- Solid inner (SI) atoms have 12 NNs within the chosen cut-off, and their PDF displays a well-defined peak at a second NN distance consistent with the one of bulk FCC Au. SI local atomic environments comprise FCC-like motifs, as well as motifs with 5-fold symmetry or icosahedral symmetry.
- Liquid inner (LI) atoms have, on average, 11 NNs. The PDF for this class of local atomic environment presents the first peak at distances lower than the one for bulk lattice and lacks a pronounced second peak in correspondence to the bulk lattice one.
- Solid high-coordination surface (SHS) atoms present, on average, eight NNs, and peaks its PDF in correspondence to the second NNs (lattice bulk).
- Liquid high-coordination surface (LHS) atoms also have eight NNs on average, yet the PDF lacks a peak at the bulk lattice.
- Solid low-coordination surface (SLS) atoms find an average of 6.9 atoms at a distance consistent with the bulk NN distance.
- Liquid low-coordination surface (LLS) atoms have, on average, 6.0 atoms at a distance lower than the bulk NN distance; furthermore, the PDF does not display any peak for the second NNs.

This unsupervised approach enables an original and data-driven definition of liquid atomic arrangements in the inner part of the NPs and at the surface. Local atomic environments in a liquid phase are all characterized by the absence of a peak of their PDF in correspondence to their bulk lattice distance (i.e. the one predicted by the reference interatomic potential). This observation holds regardless of whether they lie in the inner part or at the surface of the NP, and their coordination. Furthermore, this result confirms and rationalizes the universal signature of melting for the whole NP we recently proposed^13^.

Having discriminated in automated fashion atoms in liquid and solid environments, also as a function of their spatial location in the NP, we draw novel definitions to determine melting phase changes in the NP. To this end, we monitor the time evolution of the occurrence of liquid environments, their rate of variation and at which temperature their relative population increases above the 0.4 of the total, also as a function of their distance from the centre of mass (COM) of the NP. In the following, we refer to the melting temperature of the NP (${T}_{{{{{{{{\rm{melt}}}}}}}}}^{{{{{{{{\rm{NP}}}}}}}}}$) as the temperature at which the number of inner atoms that are identified as liquid (#LI) by the clustering algorithm displays the maximum positive derivative. This melting temperature estimation method yields equivalent results w.r.t. other well-established algorithms to calculate the melting temperature, such as the caloric curve maximum derivative and the heat capacity peak (see Supplementary Fig. 13). This observation further corroborates our trust in the clustering algorithm as a tool to characterize Au NPs melting.

### Size-dependent melting

**Fig. 2:**
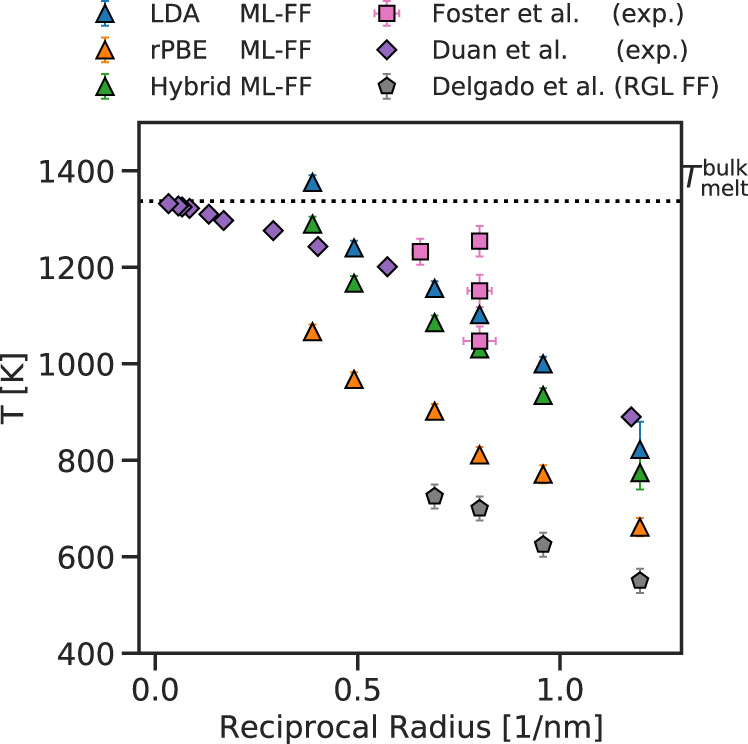
Melting temperatures of Au NPs of different sizes. Average ${T}_{{{{{{{{\rm{melt}}}}}}}}}^{{{{{{{{\rm{NP}}}}}}}}}$ as a function of NP’s reciprocal radius computed for MD simulations employing the LDA-trained (blue triangles), rPBE-trained (orange triangles) and hybrid (green triangles) ML-FFs. Experimental data for size-selected Au NPs supported on carbon (pink squares) and spherical Au NPs (purple diamonds) are taken from Foster et al.^38^ and Duan et al.^39^, respectively. Grey pentagons refer to the ${T}_{{{{{{{{\rm{melt}}}}}}}}}^{{{{{{{{\rm{NP}}}}}}}}}$ estimates from TB-SMA iterative MD melting simulations from Delgado-Callico et al.^13^. Error bars indicate the standard deviation of the melting temperature estimations, and of the NP sizes for experimental data taken from Foster et al.^38^ (pink squares).

Figure 2 reports the ${T}_{{{{{{{{\rm{melt}}}}}}}}}^{{{{{{{{\rm{NP}}}}}}}}}$ for NPs of different sizes as a function of the NPs’ reciprocal radius, as found during MD simulations carried out with the LDA, rPBE and hybrid ML-FFs. The reported ${T}_{{{{{{{{\rm{melt}}}}}}}}}^{{{{{{{{\rm{NP}}}}}}}}}$ is averaged over the four (two for NPs with more than 2500 atoms) independent MD simulations carried out for each NP and each ML-FF. For an immediate comparison, we report the experimental melting temperature of bulk FCC Au at atmospheric pressure (${T}_{{{{{{{{\rm{melt}}}}}}}}}^{{{{{\mathrm{bulk}}}}}}$), and the experimental melting temperatures of Au NPs as a function of the NP size^38,39^. For reference, we add the ${T}_{{{{{{{{\rm{melt}}}}}}}}}^{{{{{{{{\rm{NP}}}}}}}}}$ estimates obtained using a classical MD where the interatomic interaction is derived in the second-moment approximation of the tight-binding (TB-SMA)^13^. All the ML-FFs lead to ${T}_{{{{{{{{\rm{melt}}}}}}}}}^{{{{{{{{\rm{NP}}}}}}}}}$ predictions, which are (as expected) lower than the ones found during experiments for C-supported Au NPs (pink squares in Fig. 2). On average, the rPBE-derived ML-FF predicts ${T}_{{{{{{{{\rm{melt}}}}}}}}}^{{{{{{{{\rm{NP}}}}}}}}}$ 250 ± 50 K lower than the ones predicted by the LDA-derived ML-FF, and 180 ± 40 K lower than the ones predicted by the hybrid ML-FF. Interestingly, the ${T}_{{{{{{{{\rm{melt}}}}}}}}}^{{{{{{{{\rm{NP}}}}}}}}}$ predicted by the hybrid ML-FF are <50 K away from the melting temperatures found experimentally via differential scanning calorimetry measurements^39^.

### Melting mechanism characterization

In the previous section, we established the quantitative agreement between the ML-FFs’ predictions and the experimental melting temperatures of Au NPs, also as a function of their size. It is then natural to proceed further and analyse the mechanism by which phase changes occur.

**Fig. 3:**
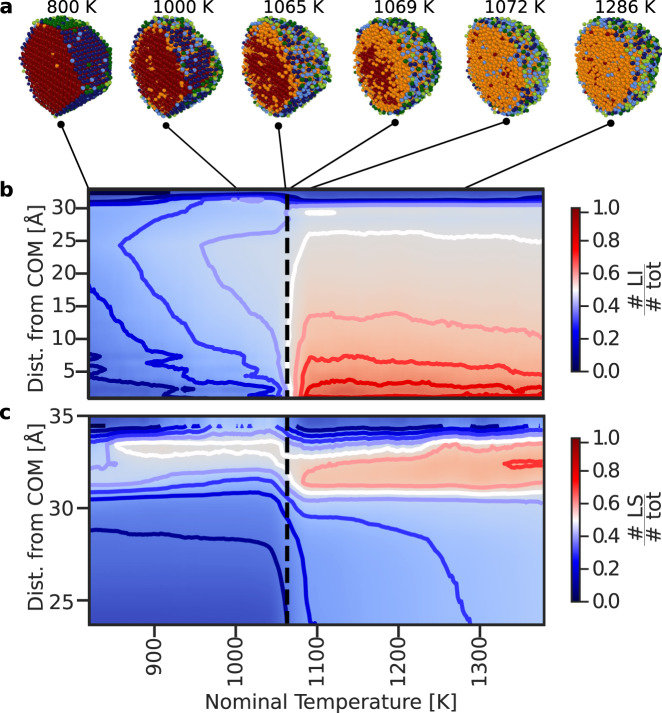
Distribution of liquid environments in an Au 6266 NP. **a** Snapshots of Au 6266 simulated using the rPBE ML-FF at different nominal simulation temperatures, with atoms coloured according to the clustering algorithm, and using the same colour scheme as in Fig. 1. **b**, **c** Average fraction of #LI (**b**) and #LS (**c**) local atomic environments as a function of the radial distance from the COM (*y*-coordinate), and of the nominal system temperature (*x*-coordinate). The bold coloured lines in (**b**, **c**) indicate the isosurfaces in the plot, from 0 to 1 every 0.1, while the black dashed line indicates the ${T}_{{{{{{{{\rm{melt}}}}}}}}}^{{{{{{{{\rm{NP}}}}}}}}}$ of 1065 K.

To this end, we display in Fig. 3 example snapshots of an Au 6266 NP at different temperatures (panel a), and the temperature-dependent radial distribution of the fraction of LI (#LI/#tot, panel b) and of LS (#LS/#tot, panel c) local atomic environments. The # symbol indicates the number of atoms belonging to a certain class, where we define: #LS = #LHS + #LLS, and #tot = #LHS + #LLS + #LI + #SHS + #SLS + #SI. The results we report are found by averaging over the set of independent MD melting simulations employing the rPBE-based ML-FF. We refer the interested reader to Supplementary Figs. 14 and 15 for the same plots for all systems with 147, 309, 561, 923, 2869 and 6266 atoms and using the three ML-FFs.

In Fig. 3 and Supplementary Figs. 14 and 15, the large majority of the local atomic environments are correctly labelled as solid (liquid) at the start (end) of each MD simulation. The average occurrence of all LI atoms increases with temperature, reaching around 0.5 at the ${T}_{{{{{{{{\rm{melt}}}}}}}}}^{{{{{{{{\rm{NP}}}}}}}}}$ independently of their distance from the NPs’ COM. Areas located few ångstrom below the NPs’ surface instead display significant abundances of LI atoms also at temperatures below ${T}_{{{{{{{{\rm{melt}}}}}}}}}^{{{{{{{{\rm{NP}}}}}}}}}$. Such observation is in line with experimental results by Foster et al.^38^, where a surface melting (${T}_{{{{{{{{\rm{melt}}}}}}}}}^{{{{{{{{\rm{surf.}}}}}}}}}$) temperature below the ${T}_{{{{{{{{\rm{melt}}}}}}}}}^{{{{{{{{\rm{NP}}}}}}}}}$ was observed for Au NPs of sizes comparable to the ones we analyse. This ${T}_{{{{{{{{\rm{melt}}}}}}}}}^{{{{{{{{\rm{surf.}}}}}}}}}$ was determined in Foster et al.^38^ by taking the average between the onset temperature for shape changes visible via aberration-corrected scanning transmission electron microscope and the highest temperature for which these did not occur.

To compare our results with available experimental data, we would like to introduce a numerical definition of ${T}_{{{{{{{{\rm{melt}}}}}}}}}^{{{{{{{{\rm{surf.}}}}}}}}}$. In analogy to the ${T}_{{{{{{{{\rm{melt}}}}}}}}}^{{{{{{{{\rm{NP}}}}}}}}}$ definition, ${T}_{{{{{{{{\rm{melt}}}}}}}}}^{{{{{{{{\rm{surf.}}}}}}}}}$ should be defined as the temperature at which a clear discontinuity appears in the temperature-dependent evolution of the abundances of liquid-like atoms at the surface of the NP. This is, however, not advisable. While the number of LI atoms has a clear and distinct positive jump—which allows us to define a ${T}_{{{{{{{{\rm{melt}}}}}}}}}^{{{{{{{{\rm{NP}}}}}}}}}$ (Supplementary Fig. 12)—the temperature-dependent evolution of the number of LS does not show such a clear first-order transition (Supplementary Fig. 17). The relative amount of LS atoms increases gradually with temperature for all NP sizes and all ML-FFs, and reaches values around 0.5 (white line in panel (c) of Fig. 3 and Supplementary Figs. 14 and 15) for atoms in the surface layer at temperatures approaching ${T}_{{{{{{{{\rm{melt}}}}}}}}}^{{{{{{{{\rm{NP}}}}}}}}}$.

**Fig. 4:**
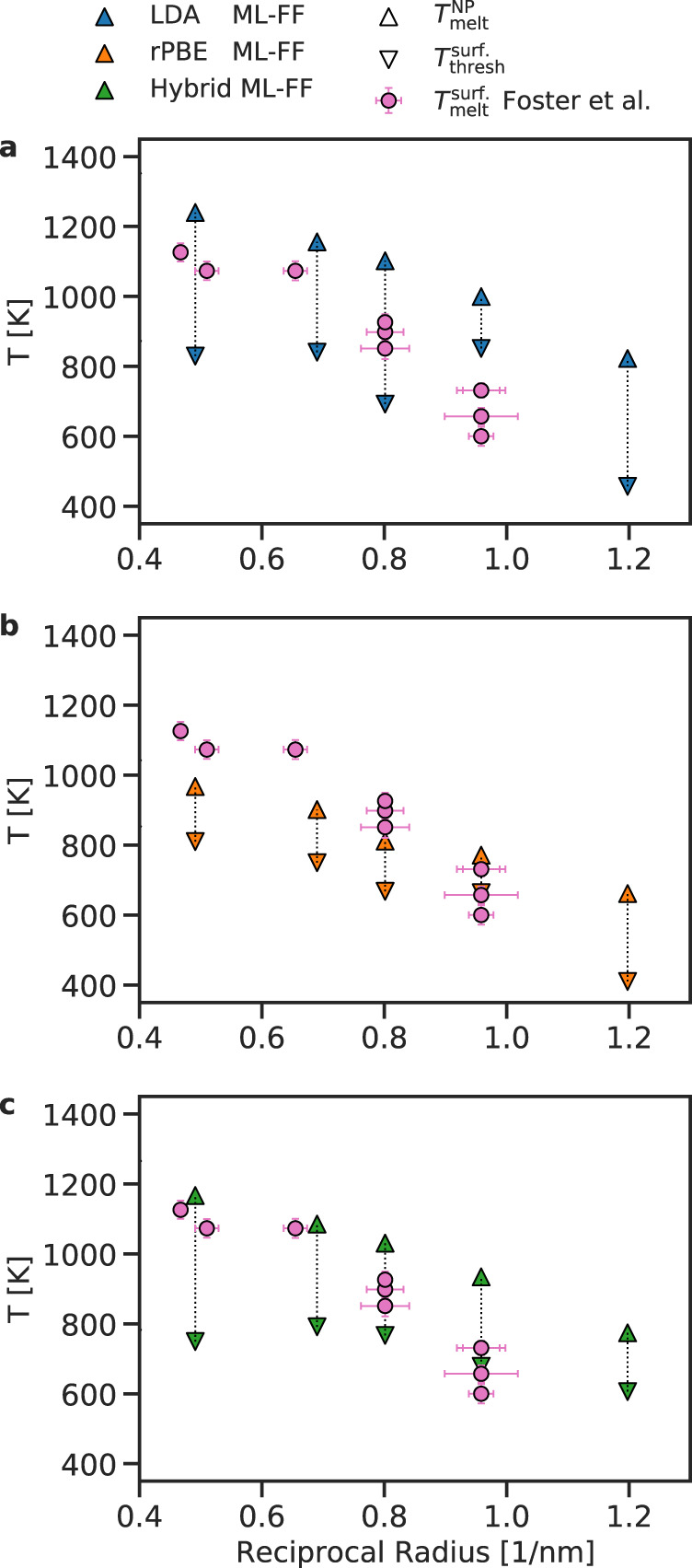
Surface phase change temperatures of Au NPs of different sizes. ${T}_{{{{{{{{\rm{melt}}}}}}}}}^{{{{{{{{\rm{surf.}}}}}}}}}$ (downward triangles) and ${T}_{{{{{{{{\rm{melt}}}}}}}}}^{{{{{{{{\rm{NP}}}}}}}}}$ (upward triangles) as a function of NPs' reciprocal radius, for MD simulations carried out using the LDA ML-FF (**a**), rPBE ML-FF (**b**), and hybrid ML-FF (**c**). Experimental estimates of ${T}_{{{{{{{{\rm{melt}}}}}}}}}^{{{{{{{{\rm{surf.}}}}}}}}}$ from high-resolution TEM measurements are taken from Foster et al.^38^, and are shown as pink circles. Error bars indicate the standard deviation of the melting temperatures and of the NP sizes for experimental data taken from Foster et al.^38^ (pink circles).

We, thus, abandon the search for an unbiased definition of ${T}_{{{{{{{{\rm{melt}}}}}}}}}^{{{{{{{{\rm{surf.}}}}}}}}}$ and introduce the quantity ${T}_{{{{{{{{\rm{thresh}}}}}}}}}^{{{{{{{{\rm{surf.}}}}}}}}}$, which provides an indication of the temperature at which significant surface rearrangement occurs. The latter is defined as the lowest temperature where at least 0.4 of the local atomic environments in the surface of the NP are classified as liquid (see the ‘Methods’ section for additional details). Figure 4 reports the values of ${T}_{{{{{{{{\rm{thresh}}}}}}}}}^{{{{{{{{\rm{surf.}}}}}}}}}$ and the ${T}_{{{{{{{{\rm{melt}}}}}}}}}^{{{{{{{{\rm{NP}}}}}}}}}$ for all our MD simulations, and the experimental ${T}_{{{{{{{{\rm{melt}}}}}}}}}^{surf.}$ as reported in Foster et al.^38^. The temperature ranges comprised between ${T}_{{{{{{{{\rm{thresh}}}}}}}}}^{{{{{{{{\rm{surf.}}}}}}}}}$ and ${T}_{{{{{{{{\rm{melt}}}}}}}}}^{{{{{{{{\rm{NP}}}}}}}}}$ are in line with the experimentally reported ${T}_{{{{{{{{\rm{melt}}}}}}}}}^{{{{{{{{\rm{surf.}}}}}}}}}$, this is especially true for the case of the hybrid ML-FF.

To deepen our understanding of the melting mechanism, we also calculate the mean first-passage temperature (MFPT) required to observe the transition to a liquid phase of 0.4 of the atoms that initially resided at an NP edge, on a (100) surface, on a (111) surface or in a bulk environment. These families of atoms are discriminated against according to their number of first NNs at the beginning of the MD simulations. Edge atoms (#NN = 6) are more likely to move into a liquid phase than atoms on a (100) facet (#NN = 8), which in turn are more prone to end into a liquid phase than the atoms on a (111) facet (#NN = 9). The inner atoms (#NN = 12) present the overall largest MFPT. This finding is coherent with the melting initiating from the outer layers of the NP (additional details are available in the ‘Methods’ and in the Supplementary Methods).

The trends observed during the melting characterization indicate that local phase changes in the outermost layer of an NP start to occur at temperatures a few hundred kelvin below the ${T}_{{{{{{{{\rm{melt}}}}}}}}}^{{{{{{{{\rm{NP}}}}}}}}}$. For Au NPs, the proposed characterization protocol establishes that local solid-to-liquid changes first initiate at low-coordinated atoms at the vertices and edges, then propagate to atoms on (100) and (111) facets, and finally proceed to the inner region of the NP.

## Discussion

We characterize the melting mechanism in gold NPs of size 1–6 nm, and predict the melting temperatures in good agreement with experimental data using molecular dynamics. These simulations employ ML-FFs, under the mapped Gaussian process framework, to surpass the trade-off between accuracy and cost in traditional atomistic modelling methods. We showcase that accurate, efficient and size-transferable FFs can be trained using small training datasets. We additionally generate a hybrid 2 + 3-body FF by linearly combining two ML-FFs fitted on data computed using different DFT functionals; this FF is parametrized to reproduce the bulk cohesive energy and yields predictions of melting temperatures of Au NPs in striking agreement with available experimental data.

To elucidate the melting mechanism, we subsequently develop a general unsupervised clustering approach to differentiate between inner and surface layers and to characterize the phase change at the atomistic level. Owing to the insight offered by the proposed clustering algorithm, we demonstrate that the melting transition initiates at the outer layer, and later spreads to the inner region. The increase in locally liquid environments in the outer region of the NP before the melting of its core finds a parallel with what is generally referred to in the literature as surface melting. The predicted trend is in very good agreement with our experimental observations, where melting was found to start at the outermost layer, at a temperature few hundred kelvin lower than the NP melting. We verify that such a melting mechanism occurs regardless of the FF used to model interatomic interactions, but we also find that different FFs predict different surface and NP melting temperatures. We expect that the data-driven simulation and characterization methods developed here, and the insight we obtain, will stimulate and benefit other research aimed at addressing the complexity of phase changes (solid-to-liquid and liquid-to-solid alike) at the nanoscale.

## Methods

### Database construction

To construct the training set, we randomly sample seven frames from a set of 60 frames extracted at regular time intervals from an ab initio MD trajectory where an Au NP containing 309 atoms (~2 nm in diameter) with an initial FCC morphology undergoes melting from 300 to 1200  K. Atomic forces and energy associated with each configuration are calculated within the DFT framework and by employing LDA and GGA-rPBE pseudopotentials to generate the training sets for the LDA and rPBE ML-FFs, respectively. The training sets we employ, therefore, contain 2163 local atomic environments and associated forces, and seven total energy values, one for each structure. When assessing learning curves (Supplementary Fig. 2) we find, in agreement with previous reports^26,27^, that the MAE on force prediction converges for training databases, which encompass a few hundreds of local atomic environments, and energy predictions do so when energies of a handful of configurations are utilized. We furthermore note (Supplementary Fig. 3) that the shape of the 2-body part of the ML-FFs resulting from training encompassing a few hundreds of local atomic environments remains, in essence, unchanged when the number of training points is increased.

We generate a validation set by extracting de-correlated frames from MD trajectories previously reported in Delgado-Callico et al.^13^, and from ab initio MD trajectories previously reported in Foster et al.^38^. We sample the melting MD trajectories reported in Delgado-Callico et al.^13^, carried out using a second-moment TB potential, from 400 up to 1200 K, and increasing iteratively the temperature of 25 K every 5 ns. For this set-up, we consider NPs containing 146, 147, 192 and 201 atoms, which present initial different closed-shell geometries, namely, octahedron (146 atoms), icosahedron (147 atoms), Marks decahedron (192 atoms), and regular-truncated octahedron (201 atoms). The NPs undergo both solid–solid and solid–liquid rearrangements during these MD trajectories (for more details see also the original reference^13^). The melting MD trajectories reported in Foster et al.^38^ are carried out via NVT simulations, as in the VASP suit, performed at temperatures from 300 to 1200 K with a 150 K interval using LDA-DFT, and for Au NPs containing 147, 309 and 561 atoms starting from a cuboctahedron.

### ML-FF construction

We construct the ML-FFs for Au by applying the framework of mappable few-body FFs trained via GPR^26,27^ using the FLARE Python Package^25,28^. GPR FFs hinge on the nearsightedness principle of quantum mechanics to predict total energies for a system of atoms *S* as a sum of local atomic energy contributions *ε*_*i*_(*ρ*_*i*_):

1 $$E(S)=\mathop{\sum}\limits_{i\in S}{\varepsilon }_{i}({\rho }_{i}),$$

where the local atomic energy is predicted as:

2 $${\varepsilon }_{i}({\rho }_{i})=\mathop{\sum}\limits_{n}k({\rho }_{i},{\rho }_{n}){\alpha }_{n}.$$

In Eq. (2), *k*(*ρ*_*i*_, *ρ*_*n*_) is the kernel (or similarity) function computed between two local atomic environments, the weights *α* are analytically calculated during the training process and *n* is the index that runs from 0 to the number of training data points employed. We employ 2- and 3-body kernels for local atomic environments, which compare local atomic environments *ρ*_*i*_ based on their distances of pairs and triplets of atoms, respectively^26,27,40^. A local atomic environment *ρ*_*i*_ is defined as the collection of relative positions *r*_*ij*_ = *r*_*j*_ − *r*_*i*_ of all atoms *j* contained within a sphere of radius *r*_cut_ centred on atom *i*. While traditional GPR FFs are faster to compute than the electronic structure methods they are trained on, they are still orders of magnitude slower than traditional parametrized FFs. The GPR FFs are therefore transformed into tabulated FFs, which retain the accuracy of the original GPR FFs while being extremely fast to compute, on par with other classical FFs. The ability to map the GPR FFs follows from the explicit 2- and 3-body nature of the representations we adopt, and takes place via spline interpolation, following the procedure introduced by Glielmo et al.^26^ and first applied to MD simulations in Zeni et al.^27^. The hyper-parameters used to train the ML-FFs are, following the notation employed in Vandermause et al.^28^, *σ*_*s*,2_ = 0.02, *l*_2_ = 0.4, *σ*_*s*,3_ = 7.0, *l*_2_ = 8.6, *σ*_*n*_ = 0.12, *r*_cut,2_ = 8.0 Å and *r*_cut,3_ = 4.5 Å.

### Hybrid ML-FFs

We generate a third ML-FF, named hybrid, by linearly combining the 2- and 3-body FFs of the ML-FFs derived from LDA and rPBE. This is done through a parameter *β* that weights the two ML-FFs so that the energy *ε*^hybrid^ for a local atomic environment, *ρ*, is:

3 $${\varepsilon }^{{{{{{{{\rm{hybrid}}}}}}}}}(\rho )=\beta {\varepsilon }^{{{{{{{{\rm{LDA}}}}}}}}}(\rho )+(1-\beta ){\varepsilon }^{{{{{{{{\rm{rPBE}}}}}}}}}(\rho ).$$

The parameter *β* is tuned to match the experimental cohesive energy of bulk Au (3.81 eV/atom) and is set to 0.61 for our ML-FF. The resulting hybrid ML-FF is a 2 + 3-body FF, and it has cohesive energy and equilibrium bulk lattice parameter that are intermediate between the LDA and rPBE ML-FFs ones, as can be seen in Supplementary Fig. 1. We remark that the generation of such hybrid ML-FF is possible because of the strictly 2 + 3-body nature of the ML-FFs employed, and because of the similar functional forms the LDA and rPBE ML-FFs display. Furthermore, the hybrid ML-FF can be easily fitted to match the experimental cohesive energy of bulk Au solely because this energy is overestimated (underestimated) by the LDA (rPBE) ML-FF.

### DFT calculation set-up

We employ training data calculated under the LDA or GGA (GGA-rPBE pseudopotentials) to the exchange-correlation term. We carry out LDA^41^ calculations using the VASP^42,43^ with projector-augmented wave pseudopotentials^44,45^. The energy cut-off of the plane-wave basis set was 240 eV, and the tolerance for self-consistency for the electronic steps was set at 10^−6^ eV. We calculate GGA-rPBE^46^ reference energies and forces using CP2K 6.1^47^. All elements are described with the DZVP-MOLOPT basis set^48^ with cores represented by the dual-space Goedecker–Teter–Hutter pseudopotentials^49^. The plane-waves cut-off is set to 500 Ry with a relative cut-off of 50 Ry. The self-consistent cycle converges when a change of < 10^−6^ eV is observed in the estimate of the system’s energy.

### Molecular dynamics calculation set-up

To study via ML-FFs the melting of Au NPs, we perform several independent MD simulations at fixed volume in periodic boundary conditions (box width = 100 Å). We employ LAMMPS^50^ as our MD engine and the FLARE^28^ add-on for calculating the energies and forces predicted by the mapped ML-FF. The temperature of the system, controlled using a Langevin thermostat with a 100 fs noise, continuously increases at a rate of 20 K/ns, with starting temperatures that range between 400 and 700 K and ending temperatures that range between 1200 and 1500 K, depending on the NP’s sizes. Newton’s equations of motions are integrated via a velocity-Verlet algorithm with a 1 fs time step for systems with < 1000 atoms, and 2 fs for systems above 1000 atoms in the case of the LDA and rPBE ML-FFs. All simulations employing the hybrid ML-FF are carried on using a 5 fs integration time step.

### Local atomic environment descriptor

We employ a local atomic density descriptor to feature each atomic environment in an NP as a function of the relative positions of the other atoms within a cut-off set to 1.75 times the average NN distance, and therefore set to 4.24 Å for simulations employing the LDA ML-FF, to 4.42 Å for simulations employing the rPBE ML-FF and to 4.30 Å for simulations employing the hybrid ML-FF. A sensitivity analysis shows that the featurisation associated with the representation is marginally affected by the choice of the cut-off radius, as long as the latter is larger than the bulk second NNs distance (see Supplementary Methods for further detail). We adopt the 2 + 3-body atomic cluster expansion representation with four radial and four angular components and employ Bessel functions of the first kind as radial basis functions^36,37,51^.

### Clustering algorithm for phase change characterization

To apply the clustering algorithm to data generated through the use of an ML-FF, we first gather 10,000 randomly chosen local atomic environment representations from among MD simulations of all NP sizes. We then run a hierarchical *k*-means clustering^52^ algorithm to group similar representations, applying two to three iterations of *k*-means clustering to partition the local atomic environment sampled during the MD simulations into the six classes described previously (additional details can be found in the Supplementary Methods).

### Melting temperature estimation

We estimate the ${T}_{{{{{{{{\rm{melt}}}}}}}}}^{{{{{{{{\rm{NP}}}}}}}}}$ as the temperature for which the maximum positive derivative of the fraction of inner atoms labelled as liquid w.r.t. the nominal simulation temperature (or, equivalently, the simulation time) is observed. The ${T}_{{{{{{{{\rm{melt}}}}}}}}}^{{{{{{{{\rm{NP}}}}}}}}}$ is commonly defined as the temperature where the highest value of the heat capacity is observed ${T}_{{{{{{{{\rm{melt}}}}}}}}}^{{{{{{{{\rm{NP}}}}}}}}}$, or as the temperature here the highest standard deviation in the total energy is found^13,53,54^. Supplementary Fig. 13 shows the striking correspondence that exists between the ${T}_{{{{{{{{\rm{melt}}}}}}}}}^{{{{{{{{\rm{NP}}}}}}}}}$ estimated using the three aforementioned methods. This result confirms that the ${T}_{{{{{{{{\rm{melt}}}}}}}}}^{{{{{{{{\rm{NP}}}}}}}}}$ estimation methods we introduce are accurate for the systems we consider and reinforces our belief that the characterization offered by our clustering method is valid.

### Surface transition temperature calculation

**Fig. 5:**
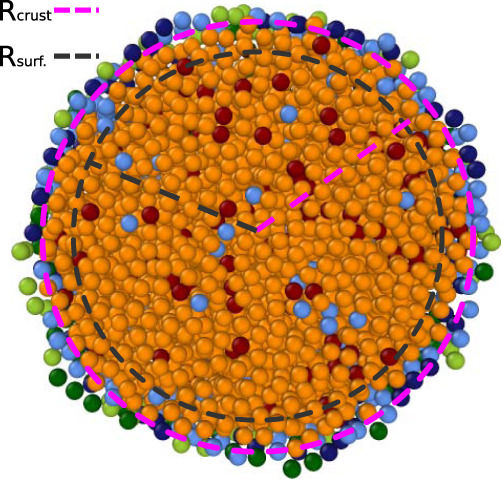
Crust and surface radiuses in an Au 6266 NP. Depiction of the values of *R*_crust_ (pink) and *R*_surf._ (grey) for an Au 6266 NP. Atoms are colour-coded according to their class as yielded by the clustering algorithm, and mirror the ones of Figs. 1 and 3.

To calculate ${T}_{{{{{{{{\rm{thresh}}}}}}}}}^{{{{{{{{\rm{surf.}}}}}}}}}$, we analyse the spatial distribution of LS atoms. We subdivide the NP in spherical shells of width 1 Å centred at the COM of the NP. We define the crust radius, *R*_crust_, as the distance from the NP COM of the spherical shell where the highest fraction of LS atoms resides. This generally coincides with the outermost radial shell of atoms in the NP. We then aim to define a surface shell and consider a second distance, *R*_surf._ = *R*_crust_ − 3 Å. The choice of a 3 Å buffer represents an arbitrary but educated guess to incorporate, approximately, a second shell of atoms in our statistics. Finally, we define ${T}_{{{{{{{{\rm{thresh}}}}}}}}}^{{{{{{{{\rm{surf.}}}}}}}}}$ as the lowest temperature at which the liquid local atomic environments in the surface of the NP amount for the 0.4 of the total number of local atomic environments in the surface shell. To exemplify the protocol, Fig. 5 displays the values of *R*_surf._ and *R*_crust_ for a snapshot extracted from an MD trajectory sampled using the rPBE ML-FF.

### Mean first-passage temperature

To evaluate the MFPT, we monitor the label assigned to each atom in the system by the hierarchical clustering scheme at each time step. The MFPT is then defined as the lowest temperature at which at least 0.4 atoms of given initial coordination are labelled as liquid environments. Since MFPTs depend on the ${T}_{{{{{{{{\rm{melt}}}}}}}}}^{{{{{{{{\rm{NP}}}}}}}}}$, for all MD trajectories we normalize each MFPT by the average ${T}_{{{{{{{{\rm{melt}}}}}}}}}^{{{{{{{{\rm{NP}}}}}}}}}$ for that particular NP size and ML-FF employed.

### Statistical information

Simulation results are obtained as averages over four independent simulations for NPs containing <1000 atoms, and over two independent simulations for NPs containing more than 1000 atoms. The ${T}_{{{{{{{{\rm{melt}}}}}}}}}^{{{{{{{{\rm{NP}}}}}}}}}$ reported for each NP size and ML-FF are the average ${T}_{{{{{{{{\rm{melt}}}}}}}}}^{{{{{{{{\rm{NP}}}}}}}}}$ computed across the four (or two) independent MD simulations. The error bars for ${T}_{{{{{{{{\rm{melt}}}}}}}}}^{{{{{{{{\rm{NP}}}}}}}}}$ reported for the *y*-axis of Fig. 2 and Supplementary Figs. 6 and 13, are calculated as the maximum between 25 K—the temperature window (see also ‘Methods’) used to individuate the peak of the positive derivative of the fraction of liquid atoms w.r.t. simulation temperature—and the standard deviation of ${T}_{{{{{{{{\rm{melt}}}}}}}}}^{{{{{{{{\rm{NP}}}}}}}}}$ computed for the four (or two) independent MD simulations for each NP size and ML-FF used to simulate them. The MAEs on energy differences (force components) reported in Supplementary Table 1 are computed on a variable number of observations, determined by the NP size, from nine (15,147) for Au 561 Co to 50 (22,050) for Au 147 Co. On average, MAEs on energy differences (force components) are calculated on 33 ± 14 (21,417 ± 12,531) samples.

## Supplementary information

Supplementary Information

## Data Availability

The tabulated Au ML-FFs, Au NPs MD trajectories and ab initio training data for Au NPs generated in this study have been deposited in the Materials Cloud database under accession code https://archive.materialscloud.org/record/2021.131^55^. Example MD trajectories are also stored in the same repository. Source data for Figs. 2 and 4 are provided with this paper. Other data are available from the authors upon request.

## Code availability

A majority of the code used in this calculation is open source. ML-FF training and mapping are carried on using FLARE (https://github.com/mir-group/flare). DFT data are gathered using CP2K (https://www.cp2k.org) and VASP (https://www.vasp.at-licencenumber5-867). MD simulations are run via LAMMPS (https://lammps.sandia.gov). The computation of local atomic environment descriptors and the clustering characterization are carried out using the Raffy Python package^56^. *K*-means clustering is done in Python via the SciPy library.
