# Supplementary material for: Data-driven simulation and characterisation of gold nanoparticle melting
Source: Nat Commun. 2021 Oct 18;12:6056. doi: 10.1038/s41467-021-26199-7 (PMC8523526; doi:10.1038/s41467-021-26199-7)
Supplement: Supplementary file 1 — Supplementary Information [file 41467_2021_26199_MOESM1_ESM.pdf]

Supplementary Information

Data-driven simulation and characterisation of gold nanoparticle melting

C. Zeni et al.

SUPPLEMENTARY FIGURES

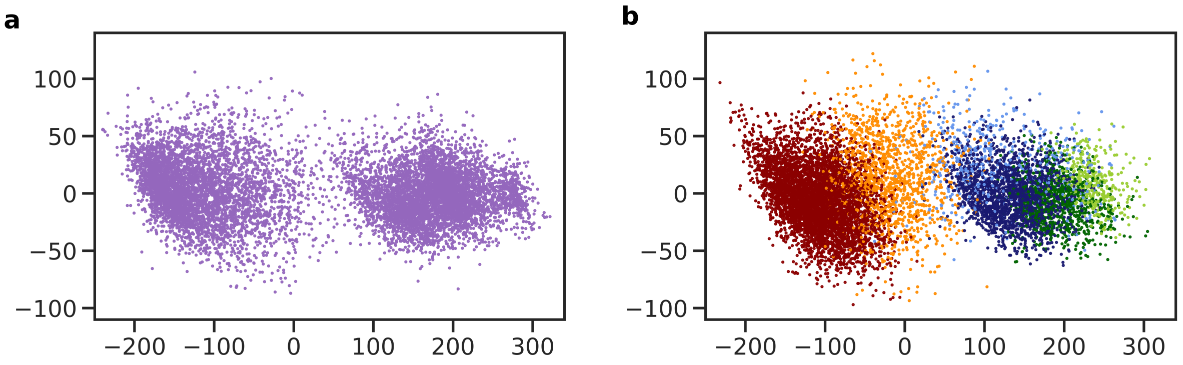

Supplementary Figure 1. **Visualization of the heterogeneity of the training dataset.** 2-dimensional PCA projection of the 40-dimensional atomic cluster expansion descriptor computed on (panel a) 10000 local atomic environments sampled from the Au 309 MD simulation used to generate the initial training set for the LDA ML-FF, and (panel b) from 10000 local atomic environments sampled from MD simulations of Au147, 309, 561, 923, 2869, and 6266 carried out using the LDA ML-FF. Points in panel b are also colour-coded according to their classification using the hierarchical k-means clustering algorithm, and following the same colour scheme as in Figure 1 and Supplementary Figures 8 and 9

.

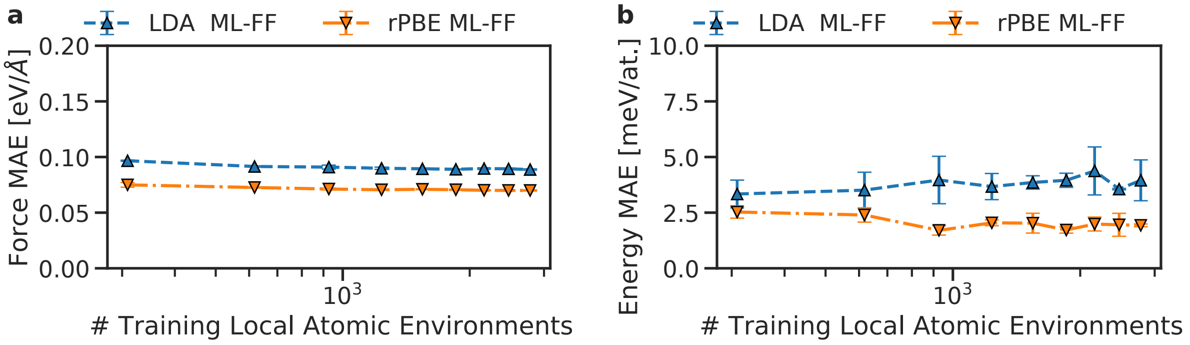

Supplementary Figure 2. **Training curves for the LDA and rPBE ML-FFs.** Test MAEs incurred by the LDA (blue) and rPBE (orange) ML-FFs on force components (panel a) and atomic energy differences (panel b), as a function of the number of local atomic environments used to train them. The error bars represent the variance of the MAEs across three independent training and testing iterations.

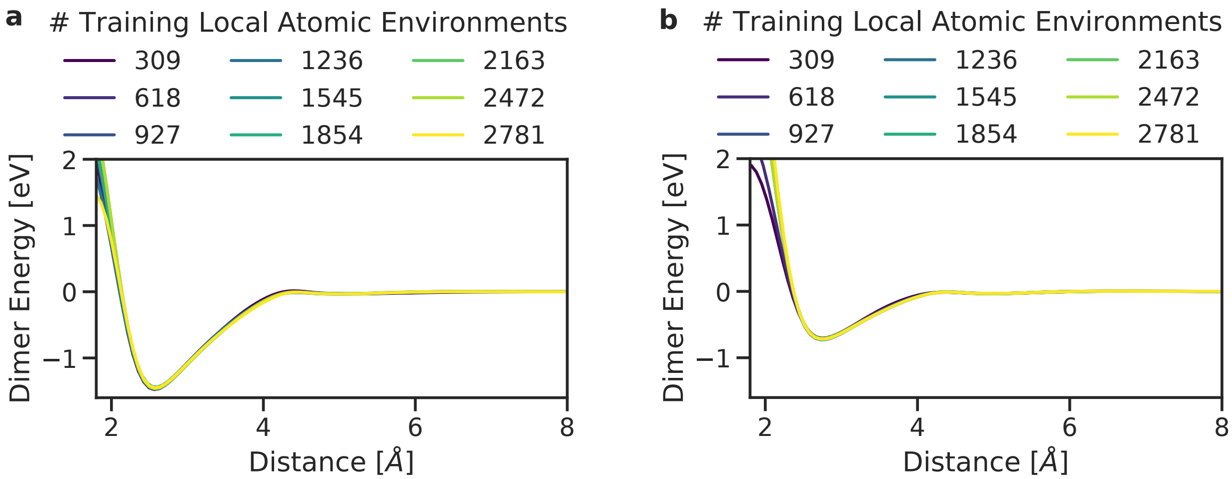

Supplementary Figure 3. **Dimer energy for LDA and rPBE ML-FFs using different training set sizes.** Dimer energy as a function of distance for LDA (panel a) and rPBE (panel b) ML-FFs trained on an increasing number of local atomic environments (color-coded, from blue to yellow).

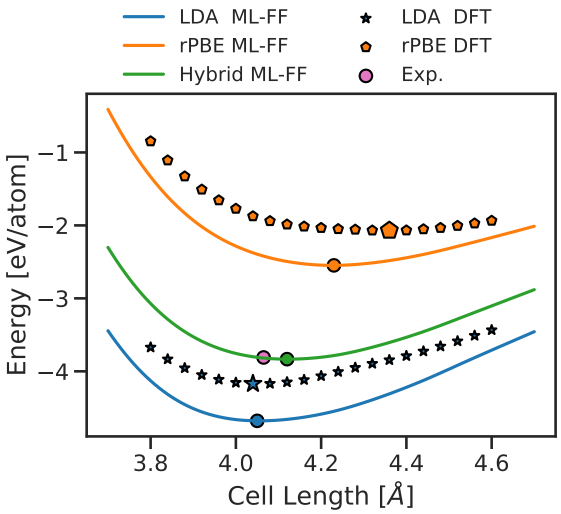

Supplementary Figure 4. **Volume-energy curves for the three ML-FFs and for LDA and rPBE ab initio calculations.** Cohesive energy per atom as a function of cell length in FCC Au computed using the LDA (blue), rPBE (orange), and hybrid (green) ML-FFs, and via ab initio calculations carried out using the LDA (blue stars) and rPBE (orange pentagons) psudopotentials used to generate the respective training sets. Blue, orange and green dots indicate the equilibrium cell length at zero pressure for the three ML-FFs, and a large orange pentagon and a large blue star indicate the same values for the DFT calculations, while a pink dot indicates the experimental cohesive energy[1] per atom and cell length[2] of FCC Au, for reference.

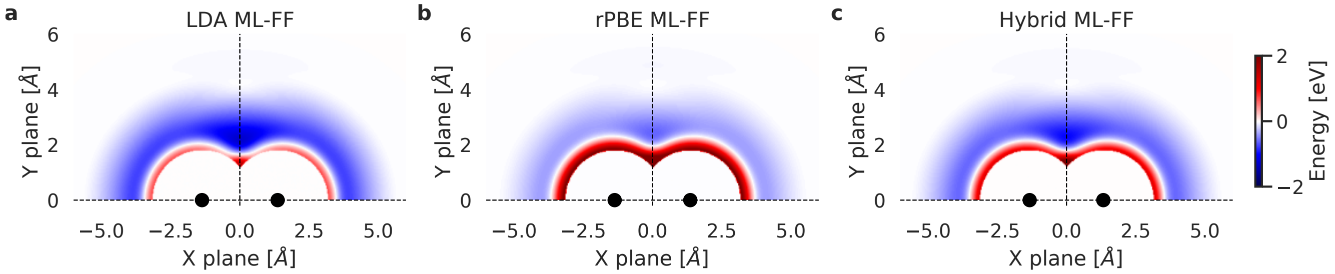

Supplementary Figure 5. **Potential energies of three Au atoms on the plane for the three ML-FFs.** Potential energy (in colour) of an Au atom neighbouring two Au atoms (black dots) as a function of its position in the x-y plane for two M-FFs trained on ab initio data extracted from LDA (a) and r-PBE (b) DFT simulations, and for the hybrid Ml-FF (c). The two neighbouring atoms are lying on the x-axis and their distance is the distance of minimum energy for an Au dimer for that FF. The potential energy felt by the Au atom is a sum of 2- and 3-body contributions, and is displayed for interatomic distances > 1.8 Å.

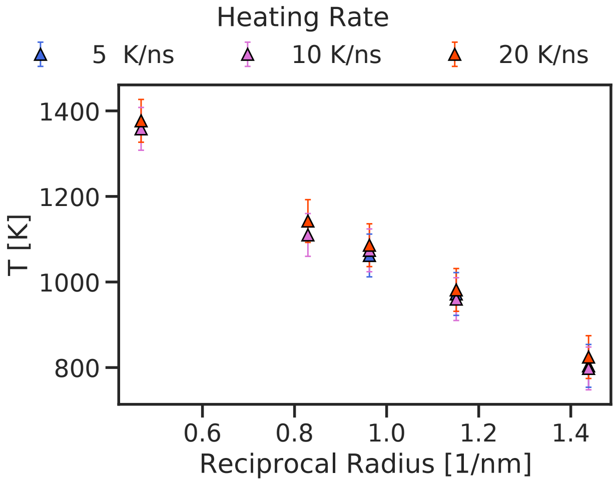

Supplementary Figure 6. **Effect of heating rate on melting temperature of Au NPs.** $T^{\mathrm{NP}}_{\mathrm{melt}}$ as a function of inverse NP radius for MD simulations of Au 147, 309, 561, 923, and 6266 carried out using the LDA ML-FF and with different heating rates (in colour). The error bars report the maximum between the standard deviation of the melting temperatures across 4 independent simulations (2 for Au 6266), and 25 K, the temperature averaging window used to estimate the melting temperature.

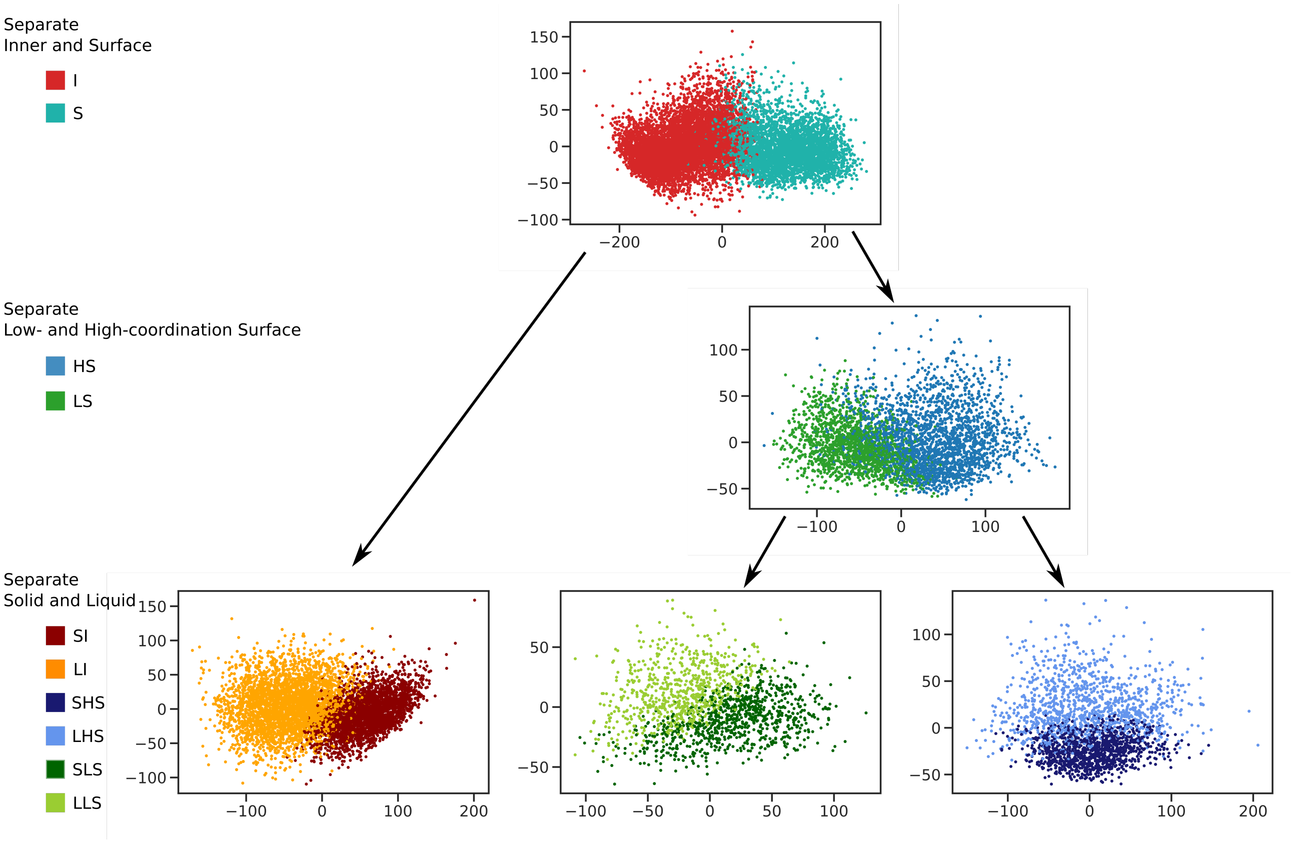

Supplementary Figure 7. **Schematic depiction of the hierarchical k-means clustering algorithm on local atomic environments.** The plots report different 2-dimensional PCA projections of 10000 local atomic environment descriptors sampled randomly from MD melting simulations of Au NPs containing 14, 309, 561, 923, 2869, and 6266 atoms carried out using the rPBE ML-FF. From top to bottom, the clustering classifies the local atomic environments as inner (bright red) and surface (green-blue). The second clustering, applied only to surface local atomic environments, is then used to distinguish between high- (blue) and low- (green) coordination local atomic environments. The third and last clustering is used to separate the liquid (light colours) from the solid (dark colours) local atomic environments, both in inner, high-coordination, and low-coordination local atomic environments.

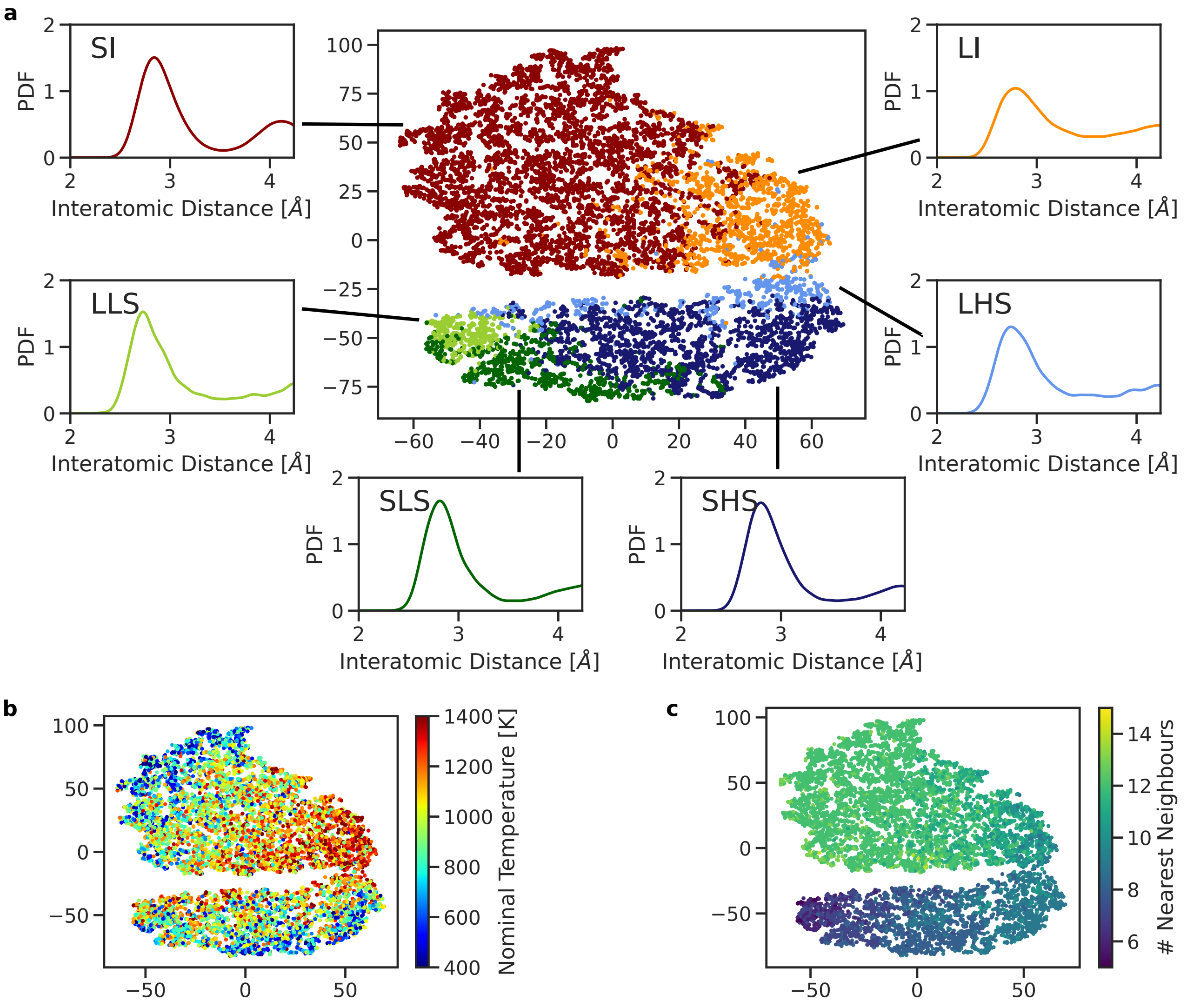

Supplementary Figure 8. **Six classes of local atoms environments identified through clustering of MD simulations using the LDA ML-FF.** Visualization of the local atomic environment representation hierarchical k-means clustering results for MD simulations of Au nanoparticles with 147, 309, 561, 923, 2869 and 6266 atoms, carried out using the ML-FF trained on LDA-DFT data. a) 1st and 2nd component (x- and y-axis) of the t-sne projection of the atomic expansion coefficients of 10^4 local atomic environments randomly sampled from melting MD simulations. The colours label the six classes assigned by the hierarchical k-means clustering algorithm, as defined in the main text. The normalized average pair-distance distribution function (PDF) belonging to each class is also reported. b), c) Same t-sne projection as in a), with colours indicating the nominal simulation temperature at which the local environment was taken from in b), and the number of nearest neighbours using a r_{cut} of 3.4 Å in c).

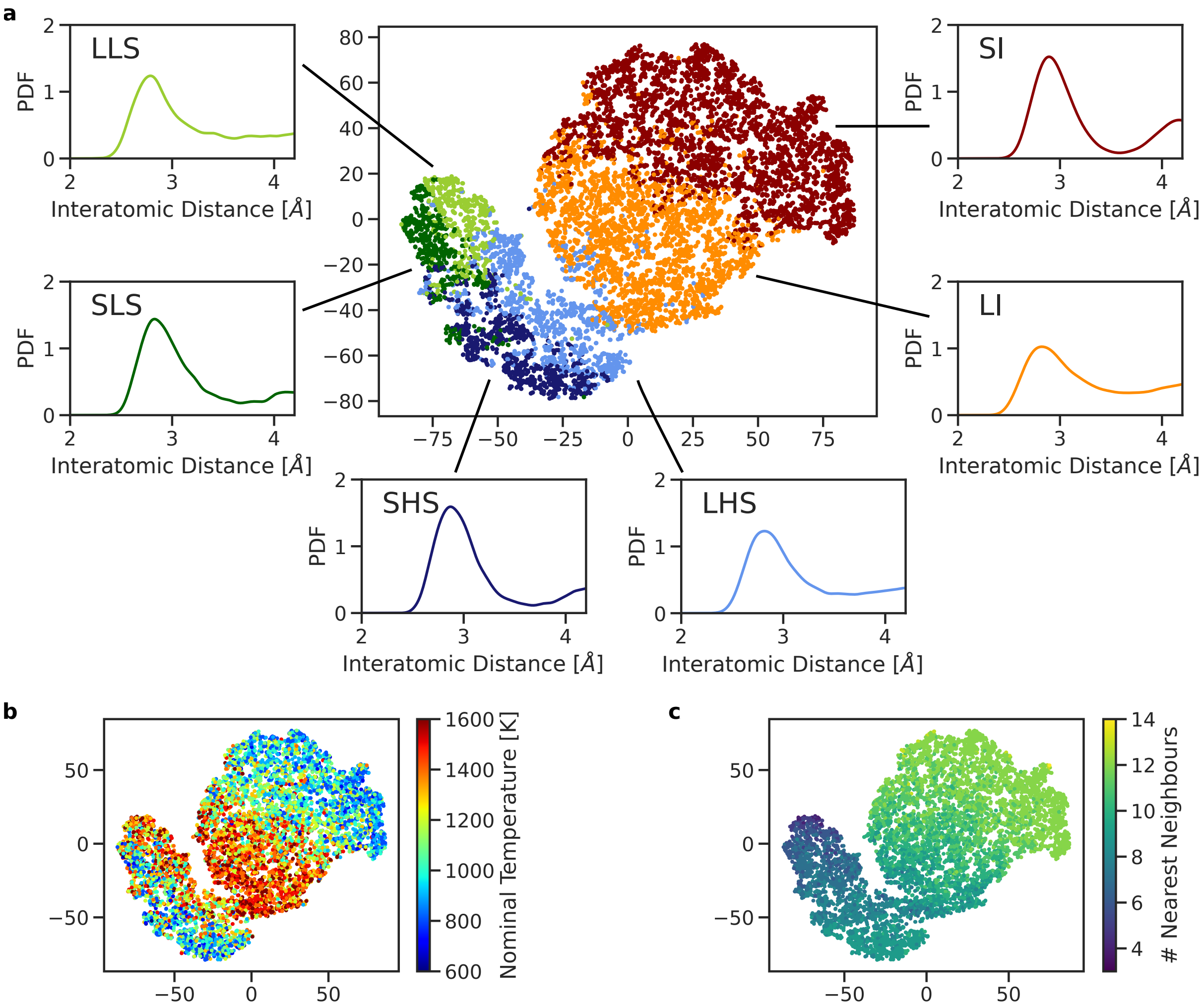

Supplementary Figure 9. **Six classes of local atoms environments identified through clustering of MD simulations using the hybrid ML-FF.** Visualization of the local atomic environment representation hierarchical k-means clustering results for MD simulations of Au nanoparticles with 147, 309, 561, 923, 2869 and 6266 atoms, carried out using the hybrid ML-FF a) 1st and 2nd component (x- and y-axis) of the t-sne projection of the atomic expansion coefficients of 10^4 local atomic environments randomly sampled from melting MD simulations. The colours label the six classes assigned by the hierarchical k-means clustering algorithm, as defined in the main text. The normalized average pair-distance distribution function (PDF) belonging to each class is also reported. b), c) Same t-sne projection as in a), with colours indicating the nominal simulation temperature at which the local environment was taken from in b), and the number of nearest neighbours using a r_{cut} of 3.4 Å in c).

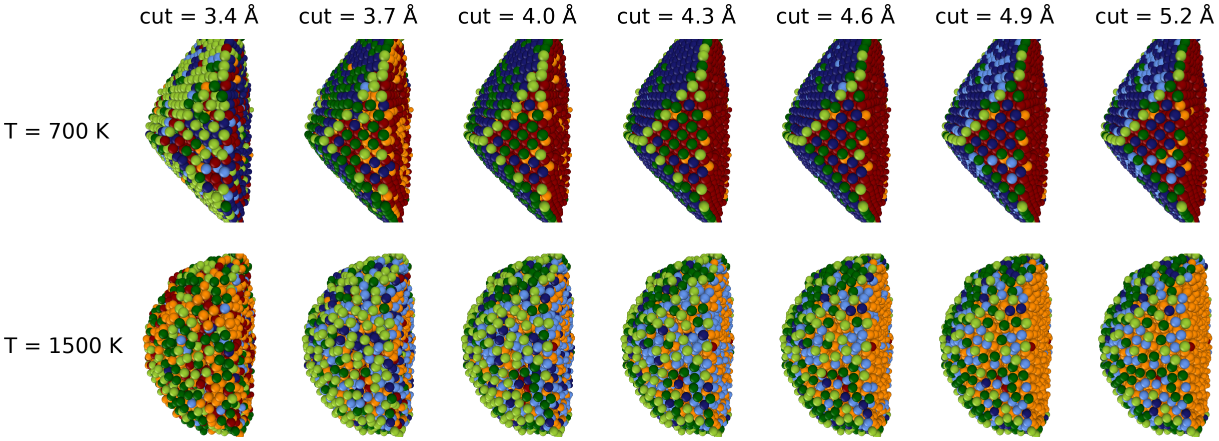

Supplementary Figure 10. **Atoms in an Au 6266 NP labeled by clustering algorithms employing different cutoff radiuses.** Two snapshots of Au 6266 taken the start (top row, T=700 K) and the end (bottom row, T=1500 K) of a MD simulation carried out using the hybrid ML-FF and coloured according to, left to right, a hierarchical clustering algorithm that employs r_{cut} of 3.4, 3.7, 4.0, 4.3, 4.6, 4.9, and 5.2 Å for the descriptor.

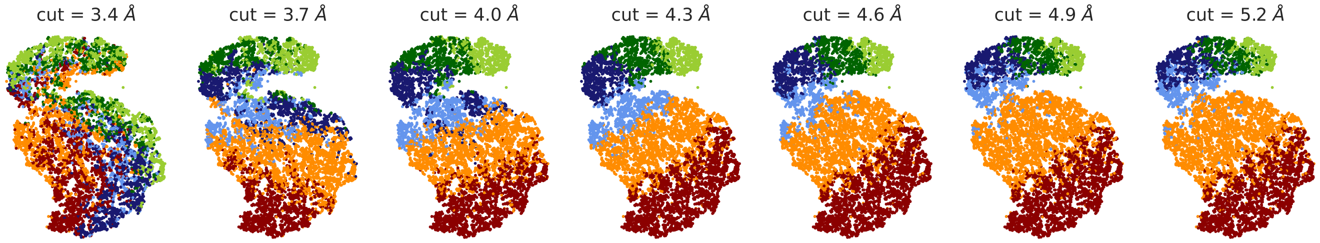

Supplementary Figure 11. **Consistency of atom labelling across clustering algorithms employing different cutoff radiuses.** t-SNE projections mirroring the ones of Figure 1 and Supplementary Figures 8 and 9, for local atomic environments taken from MD simulations carried out using the hybrid ML-FF. The x-y coordinates of points are given by the t-SNE projections of 10000 local atomic environment descriptors computed using r_{cut} = 4.30 Å. The colours label the six classes assigned by the hierarchical k-means clustering algorithms that employ different r_{cut} and mirror the ones employed and defined in the main text.

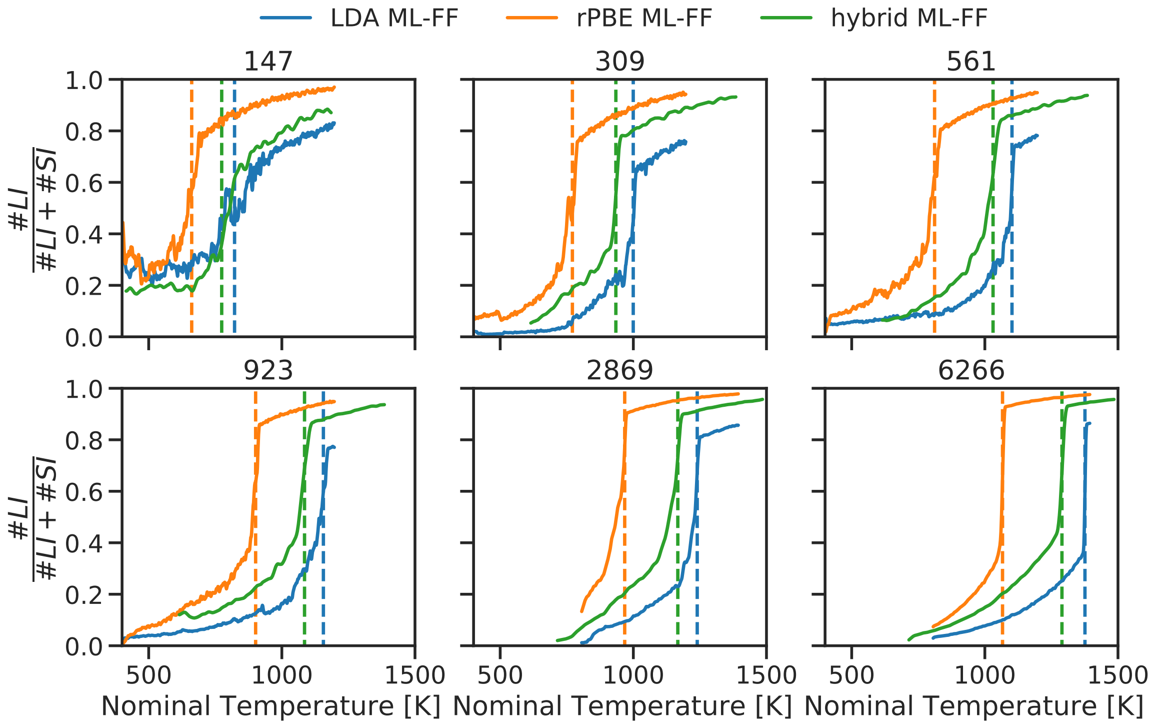

Supplementary Figure 12. **Evolution of the fraction of liquid inner atoms in MD simulations.** Fraction of inner local atomic environment that are classified as liquid by the hierarchical k-means clustering algorithm, as a function of nominal temperature for melting MD simulations carried out using the LDA ML-FF (blue), the rPBE ML-FF (orange), and the hybrid ML-FF (green). The vertical dashed lines indicate the $T_{\mathrm{melt}}^{\mathrm{NP}}$ obtained with the clustering derivative method. All lines are averaged across the repeated simulations.

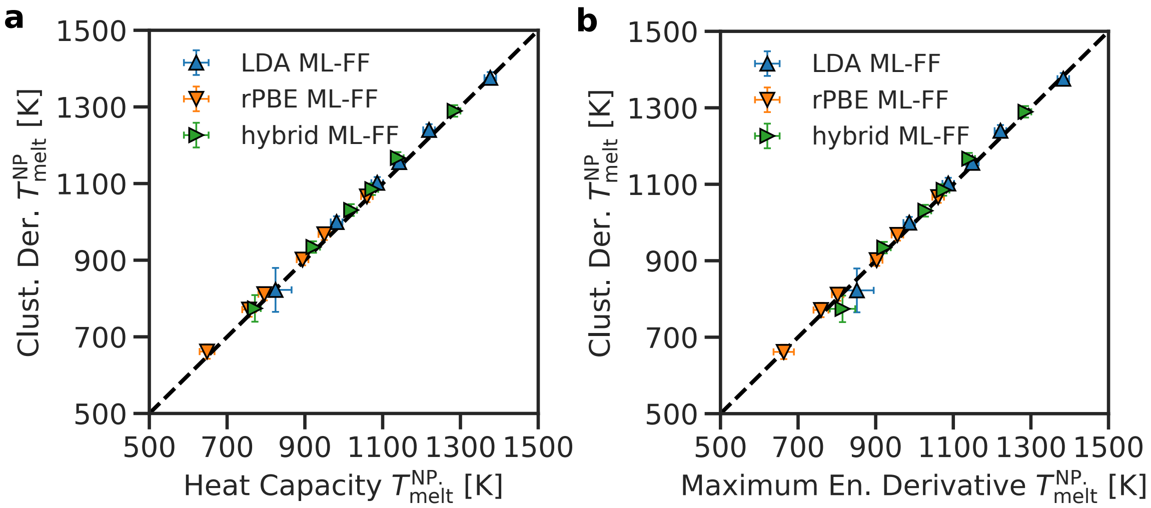

Supplementary Figure 13. **Comparison of melting temperatures using different estimation methods.** Panel a: Pairwise comparison of the $T_{\mathrm{melt}}^{\mathrm{NP}}$ for Au NPs computed as the peak of the heat capacity (x-axis) and as the peak in the derivative of the number of inner liquid clusters (y-axis). Panel b: Pairwise comparison of the $T_{\mathrm{melt}}^{\mathrm{NP}}$ for Au NPs computed as the temperature of the maximum derivative of total energy (x-axis) and as the peak in the derivative of the number of inner liquid clusters (y-axis). The error bars show the maximum between the standard deviation of the $T_{\mathrm{melt}}^{\mathrm{NP}}$ estimation across independent MD simulations, and 25 K, the temperature averaging window we employ for the $T_{\mathrm{melt}}^{\mathrm{NP}}$ calculation. The black dashed line is a visual aid that indicates a 1:1 correspondence.

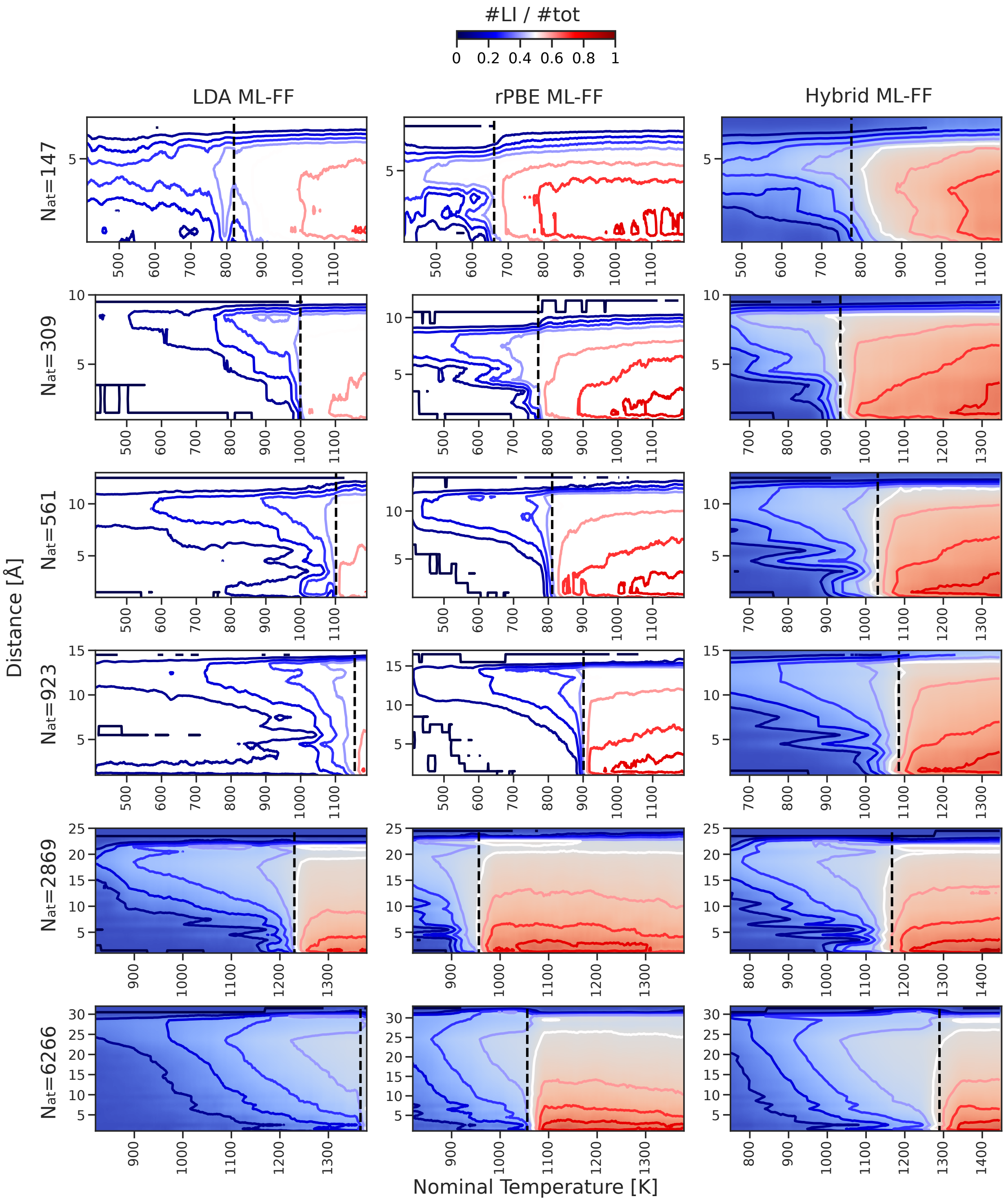

Supplementary Figure 14. **Evolution of the radial distribution of liquid inner local environments.** Average occurrence of the fraction of local atomic environments labeled as LI as a function of radial distance from the center of mass and of nominal MD simulation temperature. Plots are shown for the 6 Au NP sizes (increasing from top to bottom), and for the three ML-FFs (from left to right).

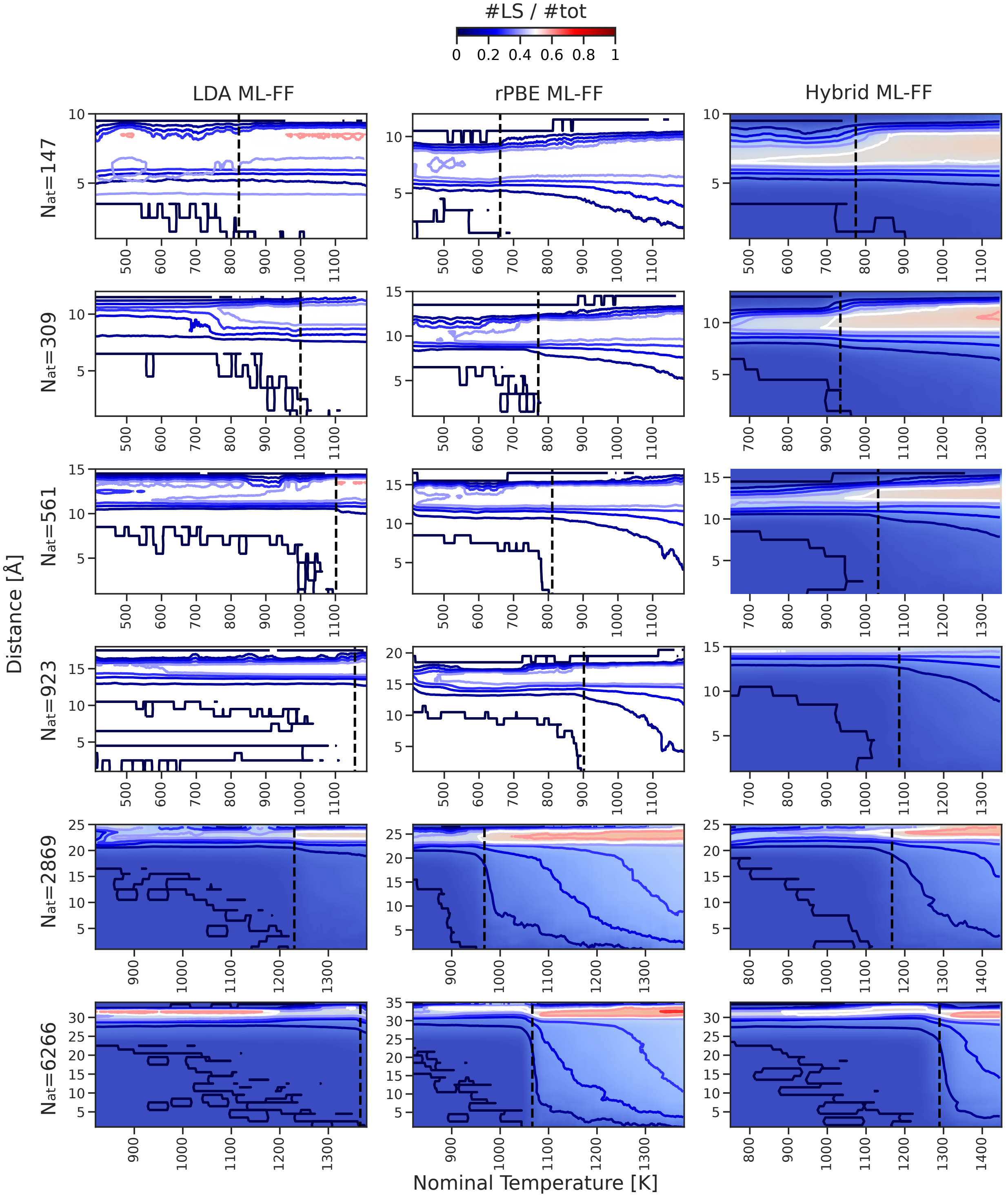

Supplementary Figure 15. **Evolution of the radial distribution of liquid surface local environments.** Average occurrence of the fraction of local atomic environments labeled as LS as a function of radial distance from the center of mass and of nominal MD simulation temperature. Plots are shown for the 6 Au NP sizes (increasing from top to bottom), and for the three ML-FFs (from left to right).

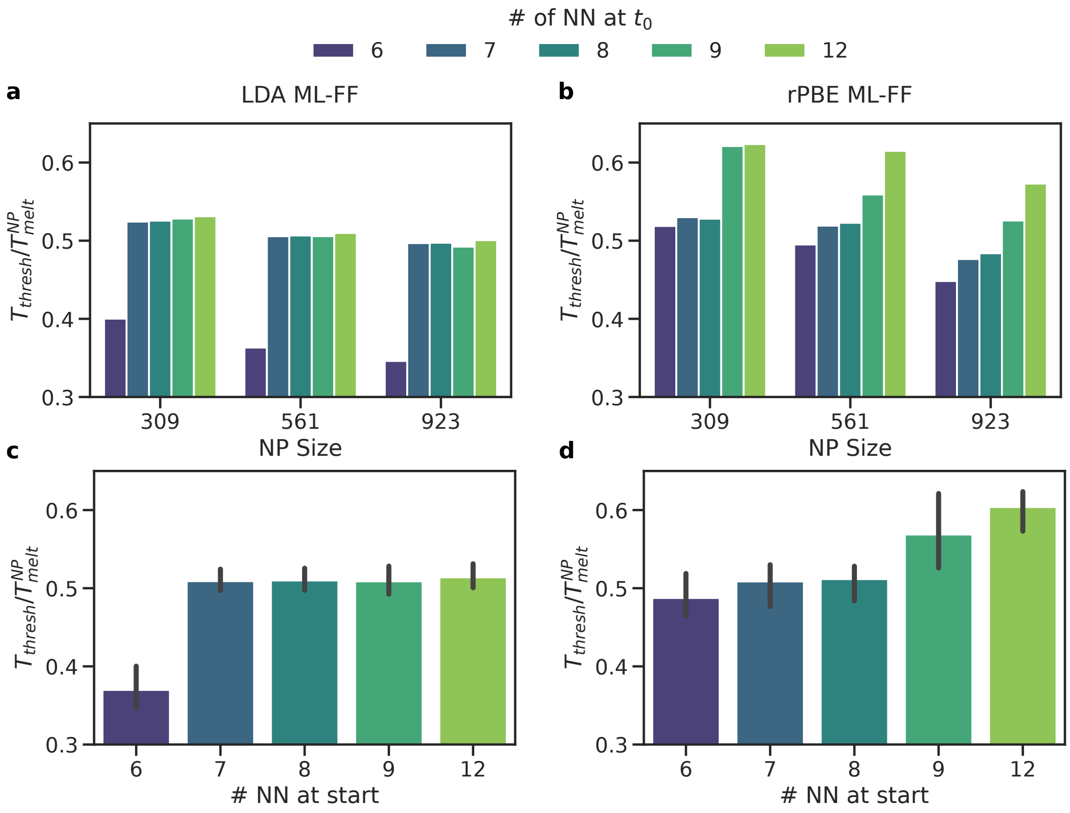

Supplementary Figure 16. **Effect of initial coordination number on melting kinetics.** Ratio between the lowest temperature where #L / #tot = 0.4 (T_{thresh}), and $T_{\text{melt}}^{\text{NP}}$ as a function of the initial number of nearest neighbours (# NN). Plots refer to MD simulations of Au 309, 561, and 923 carried out using the LDA ML-FF (panels a and c), and the rPBE ML-FF (panels b and d). Panels a and b show the behaviour of $T_{\text{thresh}}/T_{\text{melt}}^{\text{NP}}$ averaged over all MD simulations for each size considered, and grouping atoms according to their initial # NN. Panels c and d report the average and the standard deviation of the quantities of panels a and b over the three Au NP sizes considered.

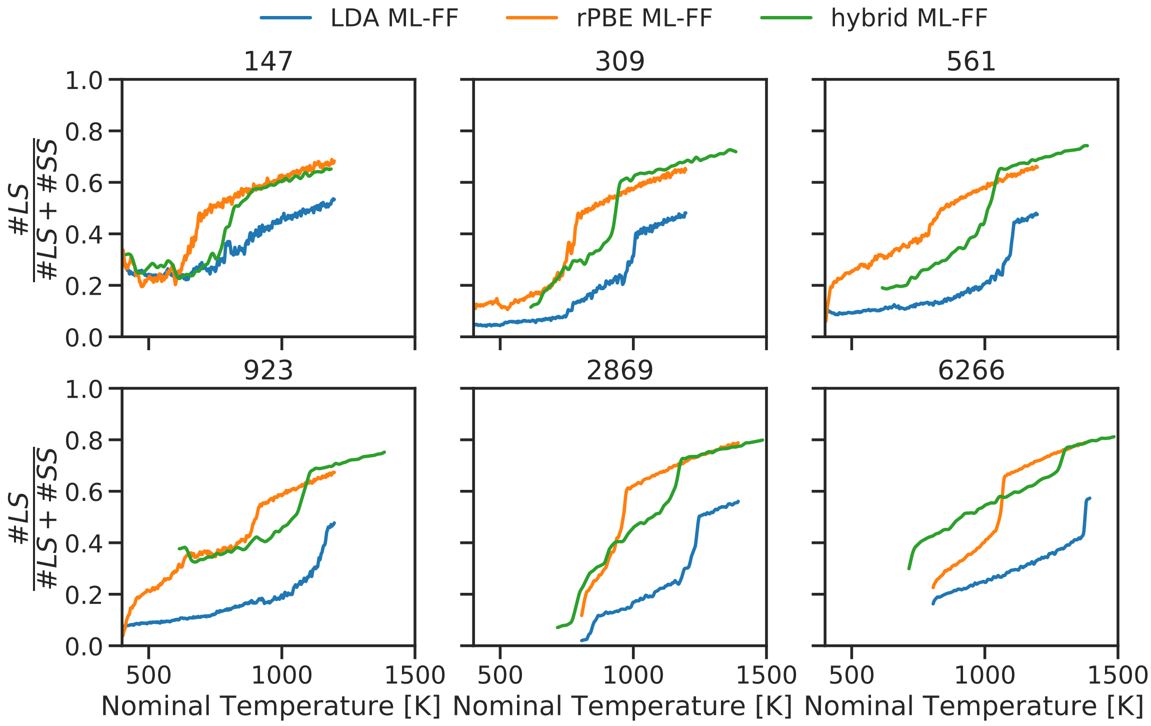

Supplementary Figure 17. **Evolution of the fraction of liquid surface atoms in MD simulations.** Fraction of surface local atomic environment that are classified as liquid by the hierarchical k-means clustering algorithm, as a function of nominal temperature for melting MD simulations carried out using the LDA ML-FF (blue), the rPBE ML-FF (orange), and the hybrid ML-FF (green).

SUPPLEMENTARY TABLES

ML-FF	Size	Shape	Force MAE [eV/Å]	Energy MAE [meV/atom]
LDA	146	Oh	0.10 ± 0.05	7.76 ± 4.90
	147	Co	0.12 ± 0.06	5.58 ± 6.00
	147	Ih	0.10 ± 0.05	10.79 ± 5.29
	192	MDh	0.10 ± 0.05	10.93 ± 5.52
	201	To	0.09 ± 0.05	9.64 ± 5.40
	**309**	**Co**	**0.09 ± 0.05**	**2.65 ± 2.02**
	561	Co	0.12 ± 0.05	2.35 ± 2.08
rPBE	146	Oh	0.09 ± 0.04	6.36 ± 3.79
	147	Co	0.10 ± 0.05	1.91 ± 1.78
	147	Ih	0.08 ± 0.04	8.40 ± 4.21
	192	MDh	0.08 ± 0.04	8.59 ± 4.10
	201	To	0.07 ± 0.04	7.28 ± 4.09
	**309**	**Co**	**0.07 ± 0.03**	**1.98 ± 1.76**
	561	Co	0.09 ± 0.04	2.31 ± 1.88

Supplementary Table I. **Validation errors on forces and energies for the three ML-FFs.** Resume of the accuracy (Force and Energy MAE) of ML-FFs trained on LDA and GGA-rPBE data, for validation sets comprising Au NPs of different sizes and shapes. We also report the standard deviation of the MAE on the same validation sets. The bold text highlights the size and shape database used to train the ML-FFs. Oh: Octahedral, Co: Cube Octahedral, To: Truncated Octahedral, Ih: Icosahedral, MDh: Marks Decahedral.

SUPPLEMENTARY METHODS

Training Database

In Supplementary Figure 1a we show a scatter plot of the first two components yielded by a principal component analysis (PCA) of the 18540 local atomic environments present in the 60 snapshots taken from a MD melting simulation of Au 309 that used to generate the training set of the LDA ML-FFs. The local atomic environments are described using the 2+3-body atomic cluster expansion representation that employs Bessel functions for the radial basis[3,4]. Supplementary Figure 1b shows the location on the same projection of 10000 randomly sampled local atomic environments observed during the extensive MD simulations we conducted in NPs of different sizes and for different temperatures employing the LDA ML-FF. We notice that the training set well covers the space spanned by the local atomic environments sampled from MD simulations and that it contains local atomic environments that belong to all six of the classes identified by the hierarchical k-means clustering algorithm.

Machine Learning Force Fields Training Curves

In Supplementary Figure 2, we report the dependency of the Mean Absolute Error (MAE) incurred by the LDA and rPBE ML-FFs trained on an increasing number of de-correlated configurations randomly extracted from an AIMD trajectory, and tested on other configurations sampled during the same MD run, and not employed for training. The test set contain approximately 15000 local atomic configurations (and forces), and approximately 50 frames (and total energies). Three independent ML-FFs were trained for each training set size, and in Supplementary Figure 2 we display the mean MAEs and their standard deviation across these three repetitions. We observe that the MAE on force components for both ML-FFs (Supplementary Figure 2a) plateaus around 10^3 training local atomic environments, corresponding to 3 Au 309 structures. The MAEs on energy differences remain confined between 2 and 4 meV/atom for the training size range, and present larger fluctuations than the force MAEs. This is expected, as only one energy reference is associated with each structure, while the force references are 309 (3-components each) per structure (one for each atom in the frame). Overall, the MAEs incurred for forces and energy differences present negligible variations above $2 \cdot 10^3$ training points; this justifies our choice of employing 7 Au 309 frames (2163 local atomic environments) for training the ML-FFs. The convergence of force and energy difference predictions for a rather contained number of data points in the training set is consistent with previous reports utilizing the same framework.[5,6]

To further showcase the small dependency of the 3-body ML-FFs on the number of training local atomic environments, we display the 2-body dimer energies in Supplementary Figure 3 for ML-FFs trained on different numbers of frames for the LDA (panel a) and rPBE (panel b) training sets. The stiffness/softness of the low energy minimum remains essentially unchanged when increasing the number of training points.

Volume-Energy Curves

In Supplementary Figure 4, we report the cell size-energy curves computed using the three ML-FFs, and the LDA and rPBE-GGA DFT methods, for a FCC lattice containing 32 Au atoms in periodic boundary conditions. We notice that the equilibrium cell length at zero pressure for the LDA ML-FF (4.035 Å) nearly matches the experimental value (4.065 Å), while the rPBE ML-FF has a larger equilibrium cell length (4.225 Å). Moreover, we notice how the LDA ML-FF is stiffer and more bound than the rPBE ML-FF; this effect can also be noticed by visualizing the 2+3-body ML-FF directly as seen in Supplementary Figure 5.

The ML predictions are in qualitative agreement with the DFT ground truth data. From a quantitative standpoint, the ML-FFs are found to underestimate (i.e. predict more negative) cohesive energies, with respect to both rPBE and LDA training sets. We nevertheless note that the ML-FFs' accuracies are acceptable on the grounds that no data of periodic systems were utilized to train the ML models. The hybrid ML-FF presents equilibrium cell energy at zero pressure that matches the experimental value; this is expected, as the hybrid ML-FF was fitted to match this experimental property.

We also calculate the bulk modulus of FCC Au at 0 K for the three ML-FFs as[7]:

$$K = \Omega_0 \left(\frac{\partial \varepsilon}{\partial \Omega^2} \right)_{\Omega_0}, \quad (1)$$

where Ω is the atomic volume, and the subscript 0 indicates the equilibrium atomic volume at 0 K. We obtain values of 203, 170, and 153 GPa for the bulk moduli yielded by the LDA, rPBE and hybrid ML-FFs, respectively, whereas the experimental value for FCC Au ranges from 142 to 220 GPa, depending on the source.[8–10]

Machine Learning Potential Energy Surfaces

Mappable (2+3)-body ML-FFs are inherently interpretable since they are equivalent to a 2+3-body tabulated potential. From the latter, it is trivial to probe whether the interatomic (2+3)-body interactions result, e.g., in a stiff or in a soft potential. In Supplementary Figure 5, we report the 2D distance map for the three ML-FFs here developed, where we notice how the LDA ML-FF has a stiff and strong interaction between atoms, while the rPBE ML-FF shows a shallower potential energy surface, which also results in weaker bond strength. The hybrid ML-FF has, as expected, a shape that is in-between the one of the LDA and rPBE ML-FF. These characteristics are reflected in the $T_{\mathrm{melt}}^{\mathrm{NP}}$ we estimate for Au NPs, as the LDA ML-FF always predicts higher $T_{\mathrm{melt}}^{\mathrm{NP}}$ values than the hybrid ML-FF, which in turn predicts higher $T_{\mathrm{melt}}^{\mathrm{NP}}$ values than the rPBE ML-FF.

Machine Learning Force Fields Validation

In Supplementary Table I, we report the validation MAE on energy differences and force components incurred by the LDA-trained and r-PBE-trained ML-FFs on datasets gathered from classical molecular dynamics (MD) trajectories previously discussed in Delgado-Callico et al.[11] and in Foster et al.[12], where nanoparticles of different sizes (leftmost column) and initial morphology (second column from the left) undergo solid-solid and solid-liquid transitions. For each NP size and each morphology, a variable number of structures has been selected at random; the total number of local atomic environments used in validation is approximately 5000 for each NP size and shape.

Effect of Heating Rate on Melting Temperature

All of the results and data we show refer to melting MD simulations where the heating rate was kept constant at 20 K/ns. Here, we briefly discuss the effect of heating rate on the $T_{\mathrm{melt}}^{\mathrm{NP}}$ for Au NPs containing 147, 309, 561, 923 and 6266 atoms due to super-heating. In Supplementary Figure 6, we report the $T_{\mathrm{melt}}^{\mathrm{NP}}$ computed for MD simulations of Au 147, 309, 561, 923, and 6266 carried out using the LDA ML-FF with a heating rate of 20 K/ns, 10 K/ns for all sizes, and also 5 K/ns for NPs with less than 923 atoms. We observe that the heating rate has little effect on the $T_{\mathrm{melt}}^{\mathrm{NP}}$; this, therefore, reinforces our belief the super-heating effects are not strongly affecting our $T_{\mathrm{melt}}^{\mathrm{NP}}$ estimates.

Clustering of Local Atomic Environments

Supplementary Figure 7 shows five 2-dimensional PCA projections of 10000 local atomic environments randomly sampled from the MD simulations of Au 147, 309, 561, 923, 2869, 6266 carried out using the rPBE ML-FF. Local atomic environments are described using the 2+3-body atomic cluster expansion representation that employs Bessel functions for the radial basis set[3,4]. More specifically, we expand the local atomic environment density into 4 radial bases and 4 angular bases and employ a radial cutoff of 4.24 (4.42, 4.30) Å for LDA (r-PBE, hybrid) ML-FF MD simulation data, resulting in a 40-dimensional representation. In the first map, the colour coding shows the label obtained by utilizing a k-means clustering with two clusters (k=2) on the full set of local environments; at this step inner from surface local atomic environments are discriminated. At the second iteration of k-means clustering, applied only to local atomic environments labelled as surface by the previous clustering step, a division between high- and low-coordinated surface environments emerges. When the final clustering iteration is carried out, solid and liquid environments in each group are discriminated against.

Clustering for LDA-trained ML-FF

In Supplementary Figure 8 we show the parallel to Figure 3 in the main text, for the case of local atomic environments sampled from MD simulations carried out using the LDA-trained ML-FF. Also in this case a clear separation of the six families of local environments arises.

Clustering for hybrid ML-FF

In Supplementary Figure 9, we show the parallel to Figure 3 in the main text, for the case of local atomic environments sampled from MD simulations, carried out using the hybrid ML-FF. Also in this case a clear separation of the six families of local environments arises.

Effect of cutoff radius on Clustering

To assess the sensitivity of our local atomic environment classification algorithm to the choice of the cutoff radius of the descriptor, we perform hierarchical clustering on data coming from MD simulations carried out with the hybrid ML-FF, and using cutoff radius (r_{cut}) values ranging from 3.4 Å to 5.2 Å, every 0.3 Å. In Supplementary Figure 10, we display two snapshots taken from the start (top row, T=700 K) and the end (bottom row, T=1500 K) of a MD simulation of Au 6266 carried out using the hybrid ML-FF, where the atoms are coloured according to hierarchical clustering schemes that employ increasing r_{cut}, from left to right. We notice that the classification is coherent for $r_{\text{cut}} > 4.0$ Å in this example. This is somehow expected, as information about the position of second neighbours is key in identifying the melting (see manuscript and[11]). In Supplementary Figure 11, we display the t-SNE plot of the local atomic environment descriptor using $r_{\text{cut}} = 4.30$ Å and labelled using hierarchical k-means clustering algorithms that employ different r_{cut} values, for atoms taken from MD simulations carried out using the hybrid ML-FF, coloured according to the class assigned by the clustering algorithm. Also in this case, the labeling is coherent for $r_{\text{cut}} > 4.0$.

Melting Temperature Estimate

In Supplementary Figure 12, we report the temporal evolution of the fraction of inner local atomic environments that are classified as liquid, averaged over all MD simulations carried out for each NP size and each ML-FF. A triangular rolling average of width 50 K was used to smooth out the data to ease the visualization, and to make the $T^{\text{NP}}_{\text{melt}}$ estimate more robust. This smoothing introduces and uncertainty, which we consider equal to the standard deviation of the triangular distribution used, i.e. 12 K. For all Au NPs except Au147 and both ML-FFs, the value of #LI / (#LI+#SI) has a sharp transition, where the majority of inner local atomic environments becomes liquid. We can numerically identify such transition, which we claim is indeed the $T^{\text{NP}}_{\text{melt}}$, as the temperature where the maximum positive derivative of #LI / (#LI+#SI) w.r.t. the nominal simulation temperature (or, equivalently, simulation time) is observed. To numerically estimate such temperature, we revert to finite differences to avoid the complications in taking derivatives of noisy data. In particular, we estimate the derivative of #LI / (#LI+#SI) at temperature T^* as the difference between its value at temperature $T^* + 25\ K$ and $T^* - 25\ K$, divided by 50 K. We refer to this method to estimate the $T^{\text{NP}}_{\text{melt}}$ as the clustering derivative method. In Supplementary Figure 12 we highlight the $T^{\text{NP}}_{\text{melt}}$ estimated using this approach using vertical dashed lines, with colours matching the ones of the #LI / (#LI+#SI) lines and identifying the different MD simulations.

In Supplementary Figure 13, we report the pairwise correspondences between the $T^{\text{NP}}_{\text{melt}}$ computed using our clustering derivative method, and two commonly used algorithms. The first, named Heat Capacity in Supplementary Figure 13, estimates the $T^{\text{NP}}_{\text{melt}}$ as the temperature where a peak in the heat capacity is observed.[11,13] The second, named Maximum En. Derivative in Supplementary Figure 13, estimates the $T^{\text{NP}}_{\text{melt}}$ as the temperature where the highest standard deviation of the total energy is observed.[11,13] The three methods yield $T^{\text{NP}}_{\text{melt}}$ predictions that align almost perfectly.

Liquid Environments Evolution

Supplementary Figures 14 and Figure 15 show the dependence on the nominal simulation temperature (x-axis) and the distance from the centre of mass (y-axis) of the fraction of LI and LS local atomic environments (colour), respectively, for all NP sizes and the three ML-FFs.

Effect of Initial Coordination Number on Melting Kinetics

In Supplementary Figure 16, we report the lowest temperatures where #L / #tot = 0.4 (T_{thresh}), normalized by the $T^{\text{NP}}_{\text{melt}}$ found for each NP by the appropriate ML-FF. The $T_{\text{thresh}}/T^{\text{NP}}_{\text{melt}}$ are computed for atoms that have been divided into groups according to the number of nearest neighbours (#NN) they possess at the start of each MD simulation (t_0). Panels a and b display the $T_{\text{thresh}}/T^{\text{NP}}_{\text{melt}}$ as a function of NP size, while the size-averaged $T_{\text{thresh}}/T^{\text{NP}}_{\text{melt}}$ are displayed in panels c and d, for the LDA (a, c) and rPBE (b, d) ML-FFs. We observe that the relative temperature at which at least 40% of atoms are labelled as liquid increases with the initial coordination number. In particular, atoms starting on the edges of the NPs (#NN=6) become liquid at significantly lower temperatures than the ones starting at the surface (#NN=7, 8, 9), which in turn reach the #L / #tot = 0.4 liquid threshold at temperatures below the ones for inner atoms (#NN=12). The mean first-passage temperature to move into a liquid phase is also dependent on the system size, as surface atoms in smaller NPs display, on average, lower MFPTs than the one found for larger NPs. The present observations hold regardless of the ML-FF utilized. Nonetheless, we notice that an rPBE-based description of the interatomic interactions translates in trajectories where the change into a liquid phase is delayed w.r.t. the case of dynamical evolution sampled via an LDA-based ML-FF.

Surface Phase Change

In Supplementary Figure 17, we report the temporal evolution of the fraction of surface local atomic environments that are classified as liquid, averaged over all MD simulations carried out for each NP size and each ML-FF. A triangular rolling average of width 50 K was used to smooth out the data to ease the visualization. We observe that, in contrast with the inner local environments of Supplementary Figure 12, the lines of Supplementary Figure 17 present small positive jumps, and often at temperatures that match the $T_{\mathrm{melt}}^{\mathrm{NP}}$. This may suggest that no first-order phase transition other than the melting phase transition at $T_{\mathrm{melt}}^{\mathrm{NP}}$ takes place in the NPs. For this reason, it is not advisable to define a surface melting temperature for the systems we simulate, but rather to evaluate the temperatures at which a sizeable fraction of the surface environments is labelled as liquid.

SUPPLEMENTARY REFERENCES

[1] C. Kittel, P. McEuen, and P. McEuen, Introduction to solid state physics, Vol. 8 (Wiley New York, 1996).
[2] W. P. Davey, Physical Review **25**, 753 (1925).
[3] R. Drautz, Phys. Rev. B **99**, 014104 (2019).
[4] C. Zeni, K. Rossi, A. Glielmo, and S. De Gironcoli, Journal of Chemical Physics **154**, 224112 (2021).
[5] A. Glielmo, C. Zeni, and A. De Vita, Physical Review B **97**, 184307 (2018), 1801.04823.
[6] C. Zeni, K. Rossi, A. Glielmo, Á. Fekete, N. Gaston, F. Baletto, and A. De Vita, Journal of Chemical Physics **148**, 241739 (2018).
[7] M. A. Meyers and K. K. Chawla, Mechanical behavior of materials (Cambridge university press, 2008).
[8] C. Kittel, Inc., Sixth edition,(New York, 1986) (2005).
[9] P. Kelly, Properties of materials (CRC Press, 2014).
[10] G. V. Samsonov, Handbook of the Physicochemical Properties of the Elements (Springer Science & Business Media, 2012).
[11] L. Delgado-Callico, K. Rossi, R. Pinto-Miles, P. Salzbrenner, and F. Baletto, Nanoscale **13**, 1172 (2021).
[12] D. M. Foster, T. Pavloudis, J. Kioseoglou, and R. E. Palmer, Nature Communications **10**, 2583 (2019).
[13] J. Chen, X. Fan, J. Liu, C. Gu, Y. Shi, D. J. Singh, and W. Zheng, Journal of Physical Chemistry C **124**, 7414 (2020).
